# Long-wavelength visible to near infrared photoluminescence from carbon-bridged styrylstilbene and thiadiazole conjugates in organic and aqueous media†

**DOI:** 10.1039/d0ra10201f

**Published:** 2021-02-03

**Authors:** Takeru Inoue, Makoto Tsurui, Hiroshi Yamagishi, Yuma Nakazawa, Naoto Hamaguchi, Shoya Watanabe, Yuichi Kitagawa, Yasuchika Hasegawa, Yohei Yamamoto, Hayato Tsuji

**Affiliations:** Department of Chemistry, Faculty of Science, Kanagawa University Tsuchiya 2946 Hiratsuka 259-1293 Japan tsujiha@kanagawa-u.ac.jp; Faculty of Engineering, Hokkaido University Kita13 Nishi8, Kita-ku Sapporo 060-8628 Japan; Department of Materials Science, Faculty of Pure and Applied Sciences, Tsukuba Research Center for Energy Materials Science (TREMS), University of Tsukuba 1-1-1 Tennodai Tsukuba Ibaraki 305-8573 Japan

## Abstract

Donor–acceptor–donor conjugates composed of electron-donating carbon-bridged styrylstilbene (COPV2) and electron-accepting thiadiazole derivatives equipped with carbazolyl (Cz) terminators, Cz-COPV2-A-COPV2-Cz (A = benzothiadiazole (BTz), naphthobis(thiadiazole) (NTz), or benzobis(thiadiazole) (BBTz)), were newly synthesized and found to serve as efficient and stable long-wavelength photoluminescent dyes in organic and aqueous media. In particular, Cz-COPV2-BBTz-COPV2-Cz showed photoluminescence in the near infrared region (895–927 nm) with a photoluminescence quantum yield (PLQY) of up to 0.19 in cyclohexane and of 0.02–0.03 in THF/water mixtures. Its analogues with weaker acceptors, Cz-COPV2-BTz-COPV2-Cz and Cz-COPV2-NTz-COPV2-Cz, showed yellow to deep-red emission in organic solvents, with PLQYs of up to 0.71 in organic solvents and 0.45 in THF/water mixtures.

## Abstract 2

Efficient long-wavelength visible to NIR-emitting materials have been synthesized by use of rigid planar styrylstilbene as a donor component.

## Graphical abstract

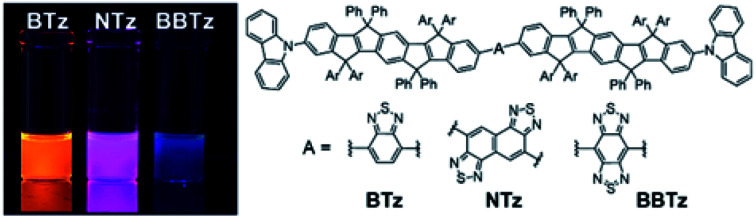

## Introduction

Long-wavelength visible to near-infrared (NIR) light-emitting materials have found a broad range of applications, in *e.g.* sensors, electroluminescence, telecommunications, and bioimaging.^1–5^ Organic NIR-emitting dyes have attracted increasing attention due to the tunability of their emission wavelength *via* molecular design, the absence of rare and/or toxic metals in their structures, and their biocompatibility, although their luminescence efficiency still requires further improvement. To achieve long-wavelength emission using such dyes, extending the π-conjugated skeleton and connecting electron-donating and -accepting groups is widely used as a design principle.^1^ However, the resulting molecules often suffer from disadvantages such as conformational disorder arising from free rotation around single bonds within the extended π-conjugated skeleton and strong intermolecular interactions that can cause aggregation, which often results in low emission efficiency by aggregation-caused quenching (ACQ).

**Fig. 1:**
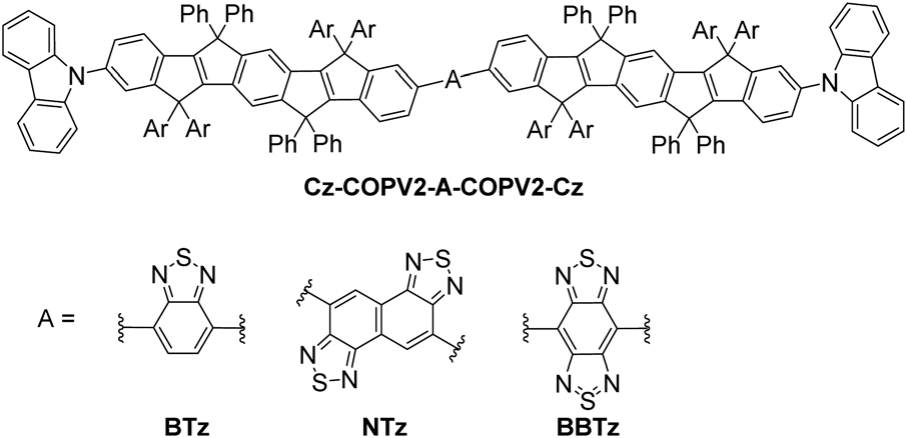
Molecular structure of Cz-COPV2-A-COPV2-Cz (A = BTz, NTz, and BBTz; Ar = *p*-octylphenyl group).

To circumvent the conformational disorder and reduce undesirable molecular motions, physical approach has been adopted in such ways as fixing molecular structures in low-temperature matrices for a long time, and more recently, aggregation-induced emission (AIE)^6–8^ and albumin-induced emission.^9–13^ Chemical construction of the structurally fixed molecules in terms of ladder-type structures that are rigidly planarized by sp^3^-carbon bridges^14,15^ and that contain bulky substituents would be an alternative methodology. To this end, we have developed carbon-bridged oligo(*p*-phenylenevinylene) (COPV),^16–18^ which features a rigid planar phenylenevinylene skeleton with bulky substituents on the bridging carbon atoms. The strong emission of COPVs in the visible region and their utility as active materials in organic lasers have been demonstrated.^19–26^ These compounds also exhibit relatively high HOMO energy levels, which make them suitable as energy donors.^27,28^ Herein, we report conjugates of carbon-bridged styrylstilbene (COPV2) and benzo- or naphtho-fused thiadiazole^29–31^ (Fig. 1) that exhibit efficient long-wavelength emission with photoluminescence quantum yields (PLQYs) of up to 0.71 in the visible and 0.19 in the NIR regions.

## Results and discussion

This desirable molecular structure was designed based on the time-dependent density functional theory (TD DFT) calculations of several model compounds at the CAM-B3LYP/6-31G* level of theory using COPV2′ (a model compound without aromatic substituents on the sp^3^-hybridized carbon atoms in the bicyclo[2.2.0]octene moieties to reduce calculation cost) and benzothiadiazole (BTz) as the donor and acceptor, respectively (Fig. S1 and Table S1†). The donor–acceptor (D–A) diad COPV2′-BTz and the triads BTz-COPV2′-BTz (A–D–A) and COPV2′-BTz-COPV2′ (D–A–D) were examined first. Among these architectures, the D–A–D triad COPV2′-BTz-COPV2′ was expected to show the longest absorption wavelength and the largest transition oscillator strengths for both absorption and emission. The installation of carbazole terminators into this compound^32–35^ to afford Cz-COPV2′-BTz-COPV2′-Cz with a D^1^–D^2^–A–D^2^–D^1^ architecture was expected to result in only a slight shift in the absorption and emission wavelengths but a significant increase in the transition oscillator strengths. Thus, a series of compounds with the carbazole-terminated architecture Cz-COPV2-A-COPV2-Cz was synthesized using BTz or the more electron-accepting naphthobis(thiadiazole) (NTz) or benzobis(thiadiazole) (BBTz) moieties as the acceptor (A).

**Scheme 1:**
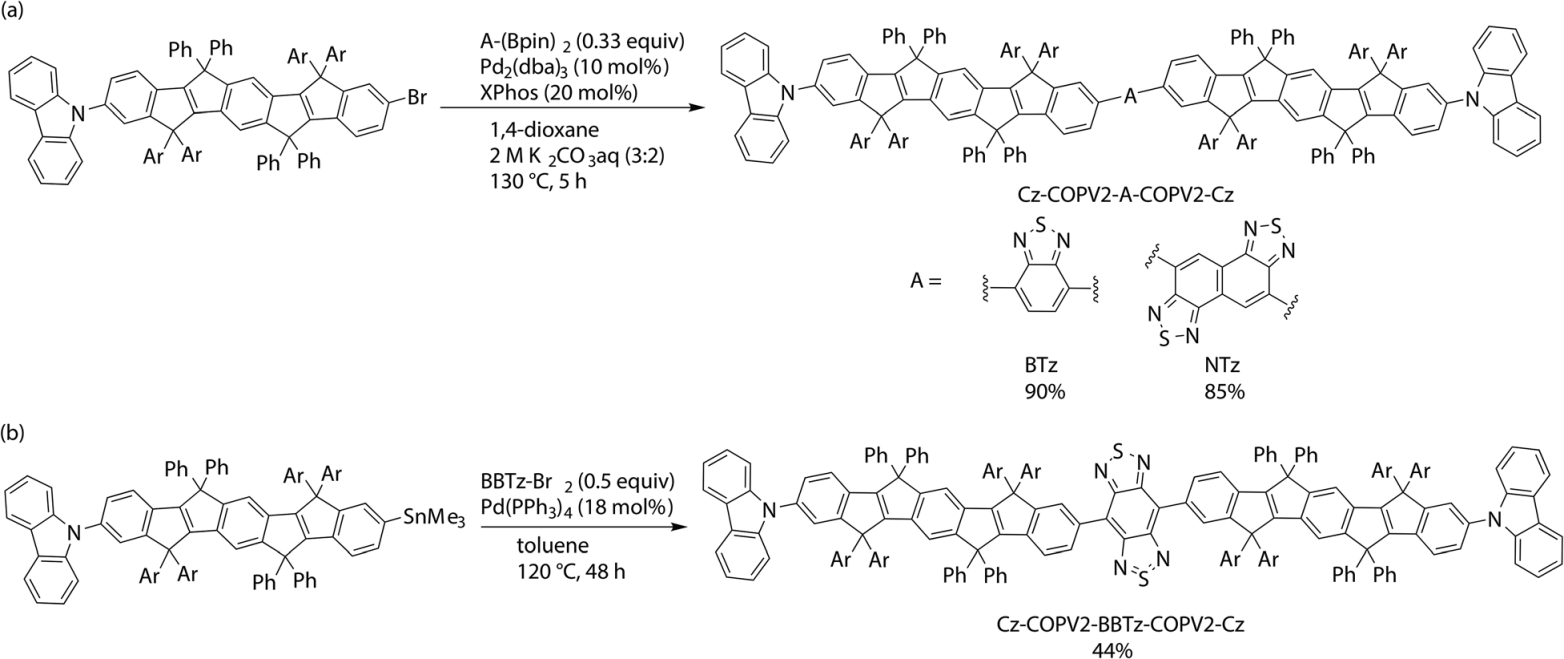
(a) Synthesis of Cz-COPV2-A-COPV2-Cz (A = BTz or NTz) by Suzuki–Miyaura coupling and (b) Cz-COPV2-BBTz-COPV2-Cz by Kosugi–Migita–Stille coupling.

The designed dyes were prepared *via* cross-coupling reactions as shown in Scheme 1. A Suzuki–Miyaura reaction between Cz-COPV2-Br and either bis(pinacolatoboryl)benzothiadiazole (BTz-(Bpin)_2_) or bis(pinacolatoboryl)naphthobis(thiadiazole) (NTz-(Bpin)_2_) afforded Cz-COPV2-BTz-COPV2-Cz and Cz-COPV2-NTz-COPV2-Cz in 90 and 85% yield, respectively.^36^ A Kosugi–Migita–Stille coupling between Cz-COPV2-SnMe_3_ and dibromobenzobis(thiadiazole) (Br-BBTz-Br)^37–43^ was suitable for the preparation of Cz-COPV2-BBTz-COPV2-Cz. The structures of the products were characterized using ^1^H and ^13^C NMR spectroscopy as well as mass spectrometry (for details, see the ESI†).

**Fig. 2:**
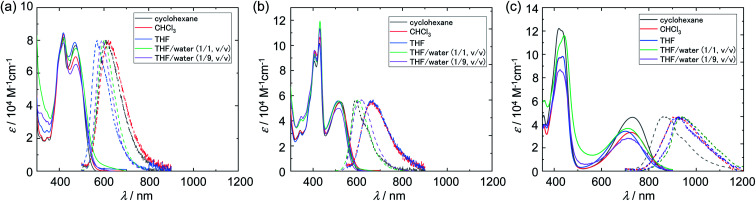
Absorption spectra (solid lines) and photoluminescence spectra (dashed lines) of Cz-COPV2-A-COPV2-Cz in different solvents; (a) A = BTz, (b) A = NTz, (c) A = BBTz.

The absorption and emission spectra in cyclohexane, THF, chloroform, and THF/water mixtures are shown in Fig. 2, and the corresponding numerical data are summarized in Table 1. Cz-COPV2-A-COPV2-Cz showed two distinct absorption bands (solid lines in Fig. 2). The shorter wavelength one at 406–442 nm with a vibronic fine structure was less sensitive to the acceptor than the other broad band, which exhibited significant bathochromic shifts depending on the electron-withdrawing ability of the acceptor group (Cz-COPV2-BTz-COPV2-Cz: 469 nm; Cz-COPV2-BBTz-COPV2-Cz: 730 nm). As suggested by calculations (*vide infra*), the former band was assigned to the transition to an excited state localized in the COPV2 moiety (LE band), while the latter was attributed to the lowest excited state with charge-transfer character from the COPV2 moieties to the acceptor (CT band, see also *E*_T_(30) plot^44^ in Fig. S2†).

**Table 1:** Summary of the photophysical properties

A	Solvent	*λ*_abs_/nm	Photoluminescence				
			*λ* a/nm	*Φ* a ^,^ b	*τ* c/ns	*k*_r_ d/10^8^ s^−1^	*k*_nr_ d/10^8^ s^−1^
BTz	Cyclohexane	416, 469	569	0.71	2.26	3.14	1.28
	CHCl_3_	420, 473	619	0.65	3.23	2.01	1.08
	THF	418, 469	607	0.70	3.02	2.32	0.99
	THF/water (1/1, v/v)	421, 475	600	0.45	2.22	2.03	2.48
	THF/water (1/9, v/v)	419, 475	593	0.37	2.05	1.80	3.07
NTz	Cyclohexane	406, 428, 522	590	0.71	2.60	2.73	1.12
	CHCl_3_	409, 430, 518	668	0.60	3.89	1.54	1.03
	THF	408, 428, 512	664	0.60	3.76	1.60	1.06
	THF/water (1/1, v/v)	408, 516	600	0.42	1.10 (0.72), 2.72 (0.28)	—	—
	THF/water (1/9, v/v)	406, 514	613	0.25	0.54 (0.79), 1.80 (0.21)	—	—
BBTz	Cyclohexane	420, 730	895	0.19^e^	1.29	1.47	6.28
	CHCl_3_	436, 725	925	0.06^e^	0.49	1.22	19.2
	THF	436, 702	927	0.04^e^	0.35	1.14	27.4
	THF/water (1/1, v/v)	442, 715	949	0.02^e^	0.40	0.55	24.4
	THF/water (1/9, v/v)	424, 710	944	0.03^e^	0.80	0.38	12.1

a Excited at each peak of the CT band.

b The photoluminescence quantum yield was determined by the absolute method for the BTz and NTz derivatives, and by the relative method for the BBTz derivatives.

c Fluorescence lifetime with excitation using a 470 nm LED for BTz and NTz, and a 630 nm LED for BBTz.

d Radiative decay constant (*k*_r_) and non-radiative decay constant (*k*_nr_) calculated from *Φ* and *τ*.

e Quantum yields were obtained using indocyanine green (ICG) in dimethylsulfoxide (*Φ* = 0.13) as a standard.^52^

Excitation at the CT band in the organic solvents afforded photoluminescence with a yellow-orange color for Cz-COPV2-BTz-COPV2-Cz (569–619 nm) and orange-red color for Cz-COPV2-NTz-COPV2-Cz (590–668 nm), depending on the solvent polarity. The photoluminescence quantum yields (PLQYs) for the CT emission were 0.60–0.71, and their fluorescence lifetimes and decay constants were typical values for fluorescence from organic compounds. The emission band of Cz-COPV2-BBTz-COPV2-Cz, which contained a strongly electron-withdrawing benzobis(thiadiazole) moiety, appeared in the NIR region (750–1200 nm). In cyclohexane, the emission peak was observed at 895 nm (PLQY: 0.19), which is relatively high for NIR emission^4,45,46^ and much higher than those of previously reported quinoidal COPV derivatives.^47,48^ The emission peak was further bathochromically shifted to 927 and 925 nm in the polar organic solvents THF and chloroform, with decreased PLQYs of 0.04 and 0.06, respectively. The fluorescence quenching in these polar solvents was also obvious in the lifetime measurements and calculated non-radiative decay constants, which obeyed the low-bandgap law.^49^

THF/water solutions were prepared by adding water to the THF solutions of the dyes. The resulting solutions of Cz-COPV2-BTz-COPV2-Cz and Cz-COPV2-BBTz-COPV2-Cz appeared to be homogeneous at micromolar concentrations and showed photoluminescence similar to that in organic solvents with a slight spectral shift. The emission peak of Cz-COPV2-BTz-COPV2-Cz was slightly hypsochromically shifted compared to that in THF [THF: 607 nm; THF/water (1/1, v/v): 600 nm; THF/water (1/9, v/v): 593 nm], and the PLQYs were moderate [THF/water (1/1, v/v): 0.45; THF/water (1/9, v/v): 0.37], with a single exponential decay profile in the fluorescence lifetime measurements. In contrast, the ‘fresh’ solution (immediately after preparation) of Cz-COPV2-NTz-COPV2-Cz in THF/water (1/1, v/v) also appeared to be homogeneous, but formed visible precipitates after 12 hours. The emission maxima of the fresh solution of Cz-COPV2-NTz-COPV2-Cz in THF/water showed hypsochromic shifts of up to 64 nm [THF/water (1/1, v/v): 600 nm; THF/water (1/9, v/v): 613 nm] relative to that in THF (664 nm). These emission maxima are similar to that in nonpolar cyclohexane (590 nm), and the fluorescence decay profile became biexponential, accompanying a short-lifetime component. These results suggest that Cz-COPV2-NTz-COPV2-Cz forms aggregates in aqueous media and that their local environment is rather hydrophobic and nonpolar,^50,51^ which seems to be a plausible origin of the drastic hypsochromic shift of the spectrum.

TD DFT calculations of model compounds at the CAM-B3LYP/6-31G* level supported the assignments of the spectral data (Table S2, Fig. S3 and S4†). The CT absorption at longer wavelength was assigned to the transition to the lowest excited state (S_1_), *i.e.*, the HOMO → LUMO transition, where HOMO is composed of two COPV2′ parts and the connecting phenylene or naphthylene moiety, and LUMO is localized within the arylenethiadiazole part. Significant redshift of the BBTz-derivative is well understood in terms of lowering the LUMO energy level (−2.38 eV for BBTz*vs.* −1.22 eV for BTz) based on the strong electron-accepting nature of the BBTz group. Calculations on the emission wavelengths were also performed using the optimized S_1_ geometry. They reproduced the experimental data well (552 nm for BTz, 568 nm for NTz, and 907 nm for BBTz) and suggests that these molecules obey Kasha's rule. The LE absorption at shorter wavelength is attributed to the transition to the S_3_ state for Cz-COPV2′-BTz-COPV2′-Cz and Cz-COPV2′-NTz-COPV2′-Cz or the S_4_ state for Cz-COPV2′-BBTz-COPV2′-Cz. There are significant contributions of the orbitals localized in the COPV2′ parts, such as HOMO−1 and LUMO+1/LUMO+2 (for BTz and BBTz) or LUMO+2/LUMO+3 (for NTz), whose orbital energy levels are rather insensitive to the acceptor part. More detailed insight into the S_1_ and S_3_ states was obtained by the geometry optimization of the excited states of Cz-COPV2′-BTz-COPV2′-Cz (Table S3†). In the S_1_ state, the bond lengths in the BTz unit (*r*_11_) and those adjacent to the BTz unit (*r*_8_ and *r*_10_) changed drastically, and the dihedral angle between BTz and COPV2 became much smaller than in the ground state (*ω*_2_: 38.48° at S_0_; 17.40° at S_1_). In the S_3_ state, the COPV unit exhibited significant structural change, whereas little change was observed in the BTz unit, suggesting an LE character of the excited state. Specifically, the bond lengths in the COPV2 unit of the excited state showed quinoidal bond alternation similar to that observed in the first excited state of the parent COPV2.

## Experimental

### Synthetic procedures

#### Cz-COPV2-BTz-COPV2-Cz

A mixture of Cz-COPV2-Br (100 mg, 61.3 μmol), 4,7-bis(4,4,5,5-tetramethyl-1,3,2-dioxaborolan-2-yl)-2,1,3-benzothiadiazole (7.8 mg, 20 μmol), Pd_2_(dba)_3_ (5.9 mg, 6.4 μmol), and XPhos (6.5 mg, 14 μmol) in 1,4-dioxane (1.1 mL) and 2 M K_2_CO_3_aq (0.7 mL) was stirred at 130 °C for 5 h under argon atmosphere. After cooling to room temperature, the reaction mixture was diluted with chloroform and passed through a short plug of silica gel. The solvent was removed under reduced pressure. The residue was purified on silica-gel column chromatography (hexane/chloroform = 3/1) to afford Cz-COPV2-BTz-COPV2-Cz (57.7 mg, 85% yield) as an orange solid. ^1^H NMR (600 MHz, CDCl_3_): *δ* 0.85–0.89 (m, 24H), 1.26–1.33 (m, 80H), 1.52–1.56 (m, 16H), 2.49–2.52 (m, 16H), 6.95–6.98 (m, 16H), 7.16–7.37 (m, 78H), 7.55 (s, 2H), 7.63 (d, *J* = 1.4 Hz, 2H), 7.81 (dd, *J* = 7.9 and *J* = 1.7 Hz, 2H), 7.91 (d, *J* = 1.4 Hz, 2H), 8.08 (d, *J* = 7.6 Hz, 4H); ^13^C NMR (150 MHz, CDCl_3_): *δ* 14.2, 22.8, 29.4, 29.6, 29.6, 29.7, 31.4, 31.5, 32.0, 35.7, 35.7, 62.7, 63.1, 63.1, 76.9, 77.1, 77.3, 110.1, 118.2, 118.3, 119.9, 120.3, 120.6, 121.2, 123.4, 123.8, 125.3, 125.9, 126.8, 126.9, 127.9, 128.3, 128.4, 128.4, 128.5, 128.5, 128.8, 133.2, 134.7, 134.9, 136.6, 136.8, 137.8, 139.1, 139.8, 140.1, 140.7, 141.5, 141.7, 143.4, 143.5, 153.8, 154.1, 154.3, 156.4, 156.6, 157.1, 157.1, 157.8, 159.2. MS (MALDI-TOF): 3236.56 [M]^+^.

#### Cz-COPV2-NTz-COPV2-Cz

90% yield. ^1^H NMR (400 MHz, CDCl_3_): *δ* 0.84–0.88 (m, 24H), 1.24–1.36 (m, 80H), 1.51–1.59 (m, 16H), 2.52 (t, *J* = 7.3 Hz, 16H), 6.97–7.01 (m, 16H), 7.17 (d, *J* = 8.2 Hz, 8H), 7.21–7.38 (m, 70H), 7.64 (s, 2H), 7.93 (d, *J* = 9.2 Hz, 2H), 8.08 (d, *J* = 7.8 Hz, 4H), 8.18 (s, 2H), 8.85 (s, 2H); ^13^C NMR (150 MHz, CDCl_3_): *δ* 14.2, 22.8, 29.4, 29.6, 29.6, 29.7, 31.4, 31.5, 32.0, 35.7, 35.7, 62.7, 62.9, 63.1, 63.1, 76.9, 77.1, 77.3, 110.1, 118.2, 118.4, 119.9, 120.3, 120.7, 121.2, 123.4, 123.8, 125.0, 125.2, 125.3, 125.9, 126.1, 126.9, 126.9, 128.3, 128.4, 128.5, 128.8, 133.9, 134.2, 134.9, 136.7, 136.8, 137.8, 139.7, 139.8, 140.0, 140.7, 141.6, 141.7, 143.4, 143.5, 153.8, 153.9, 154.0, 154.2, 156.4, 156.7, 157.1, 157.6, 157.9, 159.2. MS (MALDI-TOF): 3346.67 [M]^+^.

#### Cz-COPV2-BBTz-COPV2-Cz

A mixture of Cz-COPV2-SnMe_3_ (10.6 mg, 6.18 μmol), 4,7-dibromobenzo[1,2-*c*:4,5-*c*′]bis([1,2,5]thiadiazole) (1.1 mg, 3.1 μmol) and Pd(PPh_3_)_4_ (1.3 mg, 1.1 μmol) in toluene (1.0 mL) was stirred at 120 °C for 48 h under argon atmosphere. After cooling to room temperature, the reaction mixture was diluted with chloroform and passed through a short plug of silica gel. The solvent was removed under reduced pressure. The residue was purified on silica-gel column chromatography (hexane/chloroform = 3/1), and purified further with preparative GPC (eluent: chloroform) to afford Cz-COPV2-BBTz-COPV2-Cz (4.5 mg, 44% yield) as a green solid. ^1^H NMR (400 MHz, CDCl_3_): *δ* 0.84–0.88 (m, 24H), 1.24–1.33 (m, 80H), 1.51–1.58 (m, 16H), 2.48–2.53 (m, 16H), 6.96–6.99 (m, 16H), 7.17 (d, *J* = 8.2 Hz, 8H), 7.21–7.35 (m, H), 7.38–7.39 (m, 4H), 7.41 (s, 4H), 7.64 (s, 2H), 8.02 (dd, *J* = 8.0 and 1.6 Hz, 2H), 8.08 (d, *J* = 7.8 Hz, 4H), 8.34 (d, *J* = 1.8 Hz, 2H); ^13^C NMR (150 MHz, CDCl_3_): *δ* 14.2, 22.8, 29.3, 29.6, 29.6, 29.7, 31.4, 31.4, 32.0, 35.7, 35.7, 62.7, 62.8, 63.1, 63.1, 76.9, 77.1, 77.3, 110.1, 118.2, 118.4, 119.9, 120.3, 120.5, 121.2, 121.3, 123.4, 123.8, 125.3, 125.9, 126.9, 126.9, 128.3, 128.3, 128.4, 128.5, 128.6, 128.8, 128.8, 129.1, 130.9, 132.3, 134.9, 136.7, 136.9, 137.8, 139.6, 139.8, 140.0, 140.7, 141.5, 141.7, 143.4, 143.4, 152.8, 153.9, 154.4, 156.4, 156.8, 157.1, 157.2, 157.9, 159.2. MS (MALDI-TOF): 3295.89 [M]^+^.

### Spectroscopy

UV/vis absorption spectra were measured on JASCO V-670 or JASCO V-770. Fluorescence spectra were measured on JASCO FP-6500 for UV/vis region and a Horiba/Jobin-Yvon Fluorolog-3 spectrofluorometer equipped with a Hamamatsu R5509-43 photomultiplier for detecting NIR emissions using optical filters (excitation: SIGMAKOKI SCF-50S-58O, emission: SIGMAKOKI SCF-50S-60R) to remove higher-order diffraction of ultraviolet or visible light. Photoluminescence quantum yields for ultraviolet and visible region were measured on Hamamatsu Photonics C9920-02 Absolute PL Quantum Yield Measurement System, and absolute quantum yields were determined by using a calibrated integrating sphere system. The quantum yields in NIR region were obtained based on ICG in dimethylsulfoxide (*Φ* = 0.13) as a standard. Fluorescence lifetimes were estimated with the time correlated single photon counting (TCSPC) operation mode on Hamamatsu Photonics C11367-02.

### Calculations

Geometry optimization of the ground state of each compound was performed at the CAM-B3LYP/6-31G* level using Gaussian 09.^53^ Calculations of the excitation energy and oscillator strength, along with the geometry optimization of the excited states were performed using time-dependent (TD) DFT calculations at the CAM-B3LYP/6-31G* level.

## Conclusions

In summary, the efficacy of the use of the rigid planar electron-donating carbon-bridged styrylstilbene moiety (COPV2) to achieve efficient long-wavelength emission especially in NIR region was demonstrated in COPV2–thiadiazole conjugates. A noteworthy feature of the present chemical fixation method by intramolecular bridging is that once synthesized, the rigidity of the molecular structure is maintained even in room temperature solution independent of the solvent and without additives. The present work demonstrates that this feature played a key role in the NIR emission, which otherwise often results in low efficiency due to undesired structural disorder and molecular motion. The application of these dyes to optical devices and attempts to further elongate the spectral bathochromic shift *via* the use of longer COPV homologues and other types of acceptors are also currently under investigation in our groups, and the results will be reported in due course.

## Conflicts of interest

There are no conflicts to declare.

## Supplementary Material

RA-011-D0RA10201F-s001
