# Supplementary material for: Long-wavelength visible to near infrared photoluminescence from carbon-bridged styrylstilbene and thiadiazole conjugates in organic and aqueous media
Source: RSC Adv. 2021 Feb 3;11(11):6008–13. doi: 10.1039/d0ra10201f (PMC8694805; doi:10.1039/d0ra10201f)
Supplement: RA-011-D0RA10201F-s001 [file RA-011-D0RA10201F-s001.pdf]

Electronic Supplementary Material (ESI) for RSC Advances.
This journal is © The Royal Society of Chemistry 2021

Supporting Information

Long-wavelength Visible to Near Infrared Photoluminescence from Carbon-bridged Styrylstilbene and Thiadiazole Conjugates in Organic and Aqueous Media

Takeru Inoue,[a] Makoto Tsurui,[b] Hiroshi Yamagishi,[c] Yuma Nakazawa,[a] Naoto Hamaguchi,[a] Shoya Watanabe,[a] Yuichi Kitagawa, [b] Yasuchika Hasegawa, [b] Yohei Yamamoto,[c] Hayato Tsuji,*[,a]

a. Department of Chemistry, Faculty of Science, Kanagawa University, Tsuchiya 2946, Hiratsuka 259-1293, Japan
b. Faculty of Engineering, Hokkaido University, Kita13 Nishi8, Kita-ku, Sapporo 060-8628, Japan
c. Department of Materials Science, Faculty of Pure and Applied Sciences, and Tsukuba Research Center for Energy Materials Science (TREMS), University of Tsukuba,1-1-1 Tennodai, Tsukuba, Ibaraki 305-8573, Japan

Experimental Section

General All the reactions dealing with air- or moisture-sensitive compounds were carried out in a dry reaction vessel under a positive pressure of nitrogen or argon. Air- and moisture-sensitive liquids and solutions were transferred via syringe. Analytical thin-layer chromatography (TLC) was performed using glass plate precoated with 0.25 mm silica gel impregnated with a fluorescent indicator (254 nm). (Merck Millipore 105715). TLC plates were visualized by exposure to ultraviolet light (UV). Organic solutions were concentrated by rotary evaporation under reduced pressure. Most of the compounds were conveniently purified by silica-gel column chromatography. Flash column chromatography was performed employing Kanto Silica gel 60 (spherical, neutral, 63-210 mesh).

Materials Unless otherwise noted, commercial reagents were purchased from Wako Co., Tokyo Chemical Industry Co., Aldrich Inc. Anhydrous toluene was purchased from Wako Co. The following compound were prepared as described in the literature: 5,7,12,14-tetrahydro-5,5,12,12-tetra(*p*-octylphenyl)-7,7,14,14-tetraphenyl-di(indeno[2,1-a:2',1'-d'])-s-indacene, 3,10-dibromo-5,7,12,14-tetrahydro-5,5,12,12-tetra(*p*-octylphenyl)-7,7,14,14-tetraphenyldi(indeno[2,1-*a*:2',1'-*d'*])-*s*-indacene.[1]

Instruments Proton nuclear magnetic resonance (^{1}H NMR) and spectra were recorded using a JEOL ECA-400 (400 MHz), ECS-400 (400 MHz), or ECZ-600 (600 MHz) spectrometer. Chemical shift data for protons are reported in parts per million (ppm, δ scale) downfield from tetramethylsilane and are referenced to the residual protons in the NMR solvent ($CDCl_3$: δ 7.26). Carbon nuclear magnetic resonance spectra (^{13}C NMR) were recorded at 150 MHz: chemical date for carbons are reported in parts per million (ppm, δ scale) downfield from tetramethylsilane and referenced to the carbon resonance of the solvent ($CDCl_3$: δ 77.00). The data are presented as follows: chemical shift, multiplicity (s = singlet, d = doublet, t = triplet, m = multiplet), coupling constant in hertz (Hz), and integration. Preparative gel permeation column chromatography (GPC) was performed on a Japan Analytical Industry LaboACE LC-5060 (eluent: chloroform) with JAIGEL 2HR and 2.5HR. The MALDI-TOF MS data were obtained using a Shimadzu AXIMA® Performance in the reflection mode with dithranol or α-cyano-4-hydroxycinnamic acid as matrix.

Cz-COPV2-Br

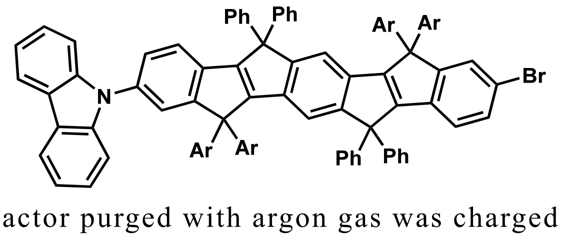

To a microwave reactor purged with argon gas was charged with **COPV2-Br$_2$** (1.50 g, 0.970 mmol), carbazole (244 mg, 1.46 mmol), $Pd_2(dba)_3$ (23.9 mg, 0.0261 mmol), tri-*tert*-butylphosphonium tetrafluoroborate (15.0 mg, 0.0517 mmol), NaO*t*-Bu (237 mg, 2.47 mmol),

and dry toluene (20 mL). The resulting solution was stirred at 150 °C for 5 min. After cooling to ambient temperature, the reaction mixture was diluted with chloroform and passed through a short plug of silica gel. The solvent was removed under reduced pressure. The residue was purified on silica-gel column chromatography (hexane/chloroform = 5:1 to 2:1) to furnish the product (543 mg, 34% yield) as a yellow solid. ^{1}H NMR (600 MHz, $CDCl_3$): δ 0.85-0.90 (m, 12H), 1.25-1.32 (m, 40H), 1.50-1.57 (m, 8H), 2.48-2.53 (m, 8H), 6.95-6.98 (m, 8H), 7.00 (d, ^3J = 8.3 Hz, 1H), 7.09 (d, ^3J = 8.3 Hz, 4H), 7.14 (d, ^3J = 8.3 Hz, 4H), 7.19-7.34 (m, 31H), 7.52 (d, ^4J = 2.1 Hz, 1H), 7.62 (s, 1H), 8.08 (d, ^3J = 8.3 Hz, 2H); ^{13}C NMR (150 MHz, $CDCl_3$): δ 14.1, 14.1, 22.7, 22.7, 29.2, 29.3, 29.5, 29.5, 29.6, 31.3, 31.9, 35.6, 35.6, 62.6, 62.6, 62.8, 63.0, 76.8, 77.0, 77.2, 110.0, 118.1, 118.2, 119.4, 119.8, 120.2, 121.1, 121.5, 123.3, 123.7, 125.2, 125.7, 126.8, 128.1, 128.2, 128.3, 128.4, 128.4, 128.5, 128.6, 130.1, 134.9, 136.4, 136.6, 137.7, 137.8, 139.2, 139.6, 140.6, 141.6, 141.7, 143.0, 143.3, 153.4, 153.8, 156.3, 156.4, 156.4, 156.9, 159.1, 159.2. MS (MALDI-TOF): 1631.19 $[M]^+$.

Cz-COPV2-SnMe$_3$

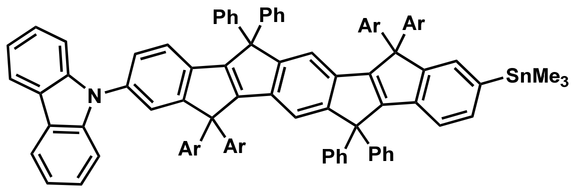

To a microwave reactor purged with argon gas was charged with **Cz-COPV2-Br** (350 mg, 0.214 mmol), hexamethyldistannane (126 mg, 0.385 mmol), $Pd(PPh_3)_4$ (50.2 mg, 43.4 μmol), dry toluene (2.0 mL). The resulting solution was stirred at 180 °C for 1 h. After cooling to ambient temperature, the reaction mixture was diluted with chloroform and passed through a short plug of silica gel. The solvent was removed under reduced pressure. The residue was purified with preparative GPC (eluent: chloroform) to furnish **Cz-COPV2-SnMe$_3$** (308 mg, 84% yield) as a yellow solid. ^{1}H NMR (600 MHz, $CDCl_3$): δ 0.18 (s, 9H), 0.85-0.90 (m, 12H), 1.25-1.33 (m, 40H), 1.51-1.57 (m, 8H), 2.49-2.52 (m, 8H), 6.95-6.97 (m, 8H), 7.12-7.16 (m, 9H), 7.18-7.34 (m, 31H), 7.55 (s, 1H), 7.62 (d, ^4J = 1.4 Hz, 1H), 8.08 (d, ^3J = 7.6 Hz, 2H); ^{13}C NMR (150 MHz, $CDCl_3$): δ -9.3, 14.1, 14.1, 22.7, 22.7, 29.2, 29.3, 29.5, 29.5, 29.6, 31.3, 31.3, 31.9, 35.6, 35.6, 62.5, 62.6, 62.9, 63.0, 76.8, 77.0, 77.2, 110.0, 118.1, 118.1, 119.8, 120.2, 120.2, 121.0, 123.3, 123.7, 125.2, 125.7, 126.7, 126.8, 128.2, 128.3, 128.3, 128.7, 132.0, 134.3, 134.7, 136.2, 136.9, 137.8, 138.9, 139.3, 139.7, 140.2, 140.6, 141.3, 141.6, 143.4, 143.4, 153.5, 154.5, 155.8, 156.2, 156.3, 156.5, 157.0, 159.1. MS (MALDI-TOF): 1715.66 $[M]^+$.

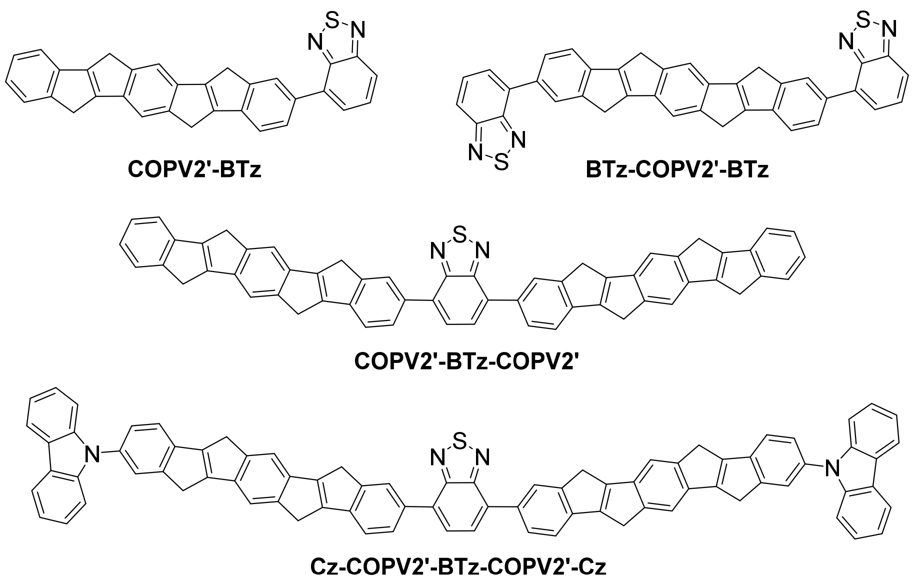

Figure S1. Structures of model compound used in calculations.

Table S1. Estimated absorption and emission wavelengths and oscillator strengths (f) of the first transitions of the model compounds calculated at TD CAM-B3LYP/6-31G* level.

Model compound	Absorption	Emission
**COPV2'-BTz**	3.2685 eV (379.33 nm)<br>f = 1.1819	2.6371 eV (470.15 nm)<br>f = 1.1054
**BTz-COPV2'-BTz**	3.1782 eV (390.11 nm)<br>f = 1.8657	2.6760 eV (463.32 nm)<br>f = 2.1847
**COPV2'-BTz-COPV2'**	2.9733 eV (416.99 nm)<br>f = 1.9168	2.2551 eV (549.78 nm)<br>f = 1.6444
**Cz-COPV2'-BTz-COPV2'-Cz**	2.9761 eV (416.59 nm)<br>f = 2.4588	2.2455 eV (552.14 nm)<br>f = 1.9380

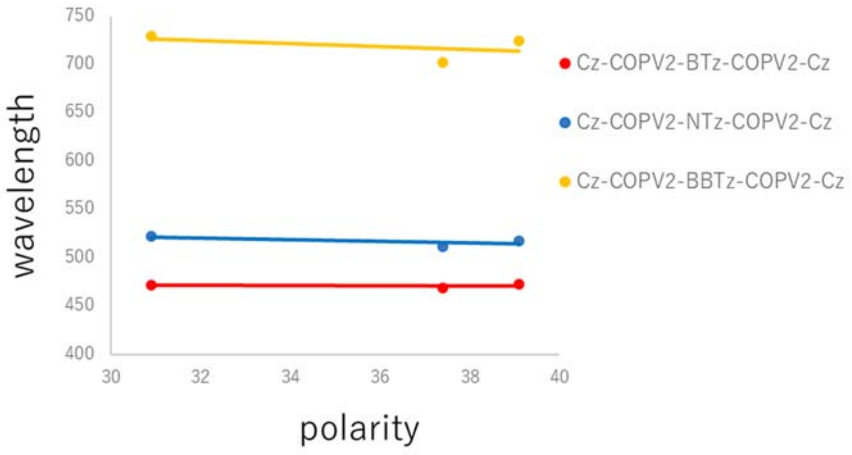

Figure S2 $E_T(30)$ plots of **Cz-COPV2-A-COPV2-Cz** (A = BTz, NTz, and BBTz)

Table S2 Summary of the calculated transition energy and oscillator strengths of model compounds **Cz-COPV2'-A-COPV2'-Cz** at the TD CAM-B3LYP/6-31G* level

A	Excited state, symmetry	Electronic configuration and coefficients [a]		Excitation (absorption) energy oscillator strength (f)	Emission energy and oscillator strength (f)[b]
BTz	S_1, B	H → L	0.558	2.98 eV (417 nm) f = 2.46	2.25 eV (552 nm) f = 1.94
	S_2, A	H−1 → L<br>H−1 → L+1<br>H → L+2	0.415<br>0.314<br>0.385	3.44 eV (361 nm) f = 0.08	
	S_3, B	H−1 → L+2<br>H → L+1	0.388<br>0.470	3.57 eV (347 nm) f = 1.92	
NTz	S_1, B	H → L	0.545	2.75 eV (450 nm) f = 1.91	2.18 eV (568 nm) f = 1.92
	S_2, A	H−1 → L	0.550	3.25 eV (382 nm) f = 0.05	
	S_3, B	H−1 → L+3<br>H → L+2	0.374<br>0.456	3.50 eV (354 nm) f = 2.56	
BBTz	S_1, B	H → L	0.621	1.84 eV (674 nm) f = 0.97	1.37 eV (907 nm) f = 0.94
	S_2, A	H−1 → L	0.623	2.57 eV (482 nm) f = 0.02	
	S_3, B	H−4 → L<br>H−2 → L	0.439<br>0.361	2.95 eV (420 nm) f = 0.19	
	S_4, B	H−1 → L+2<br>H → L+1	0.393<br>0.514	3.44 eV (360 nm) f = 3.27	

[a] H: HOMO, L: LUMO. Major contributions are listed. [b] Single point calculations were performed using the optimized S1 geometry.

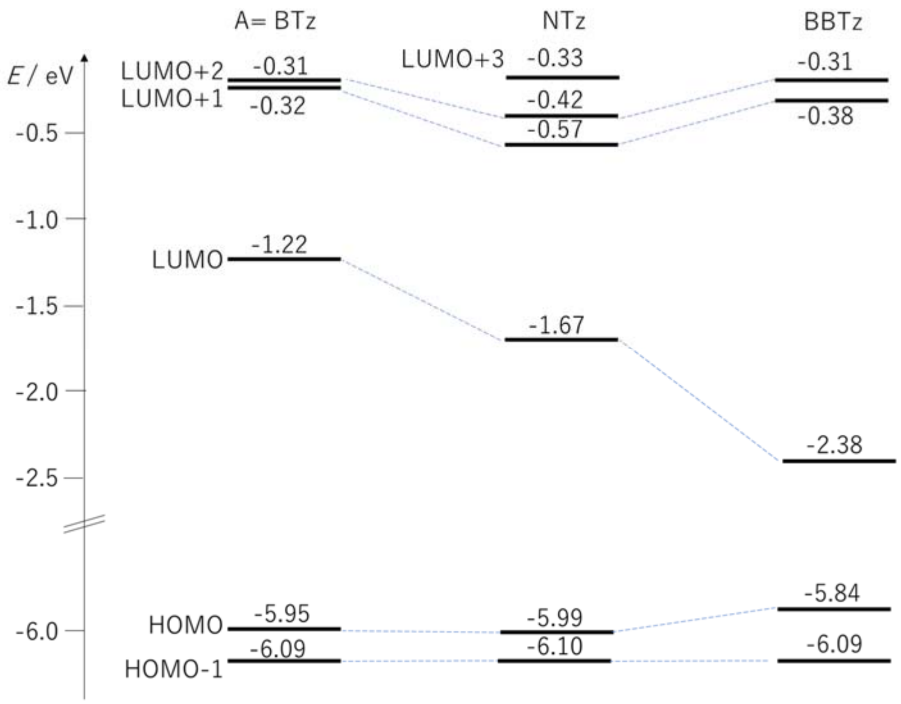

Figure S3 Orbital energy levels of model compounds **Cz-COPV2'-A-COPV2'-Cz** calculated at the CAM-B3LYP/6-31G* level

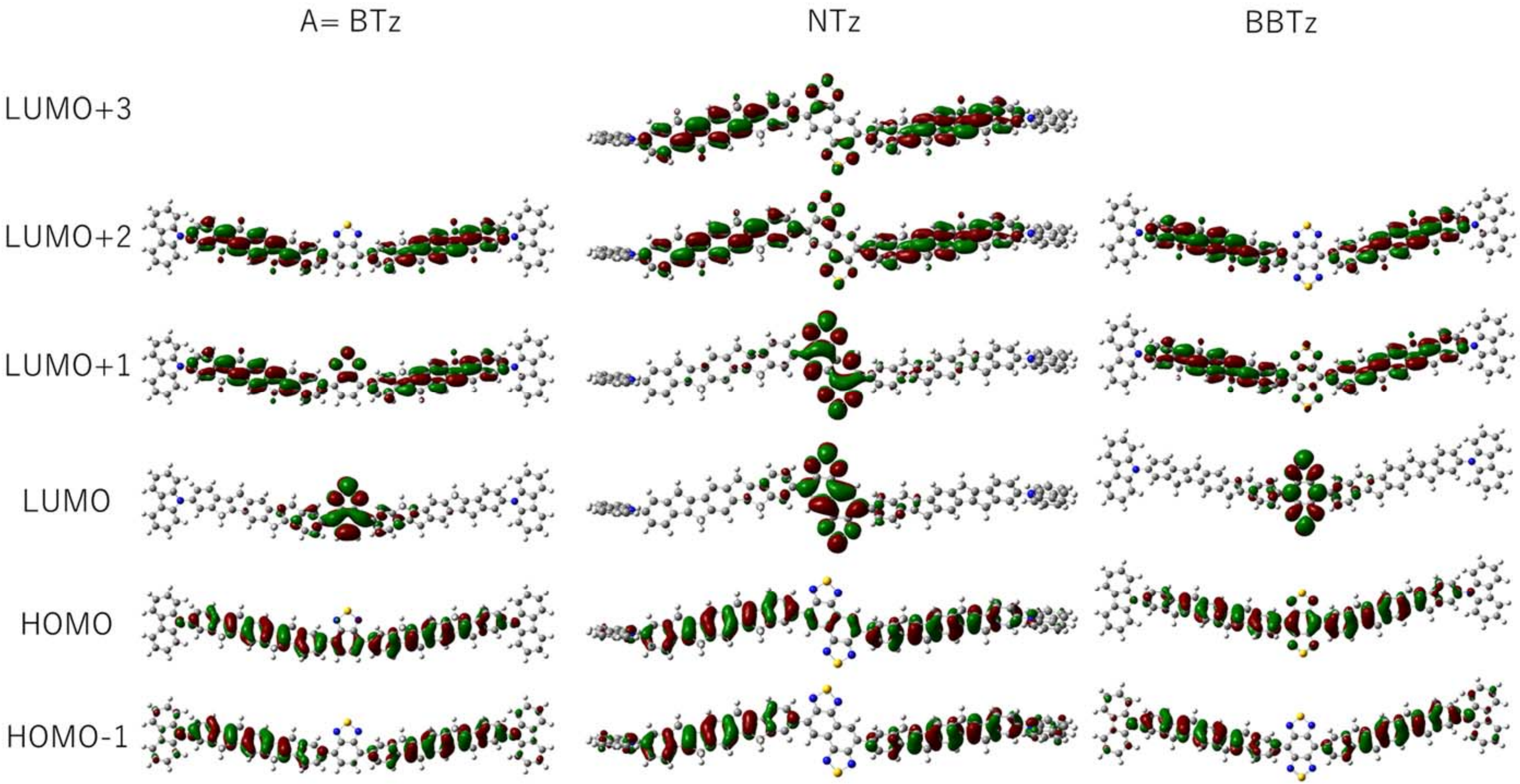

Figure S4 Kohn-Sham orbitals of model compounds **Cz-COPV2'-A-COPV2'-Cz** using 6-31G* basis set.

Table S3. Representative geometrical parameters of optimized structures of model compound **Cz-COPV2'-BTz-COPV2'-Cz** at TD CAM-B3LYP/6-31G* level. Percentage in the parentheses are change of bond length from the ground state (S_0).

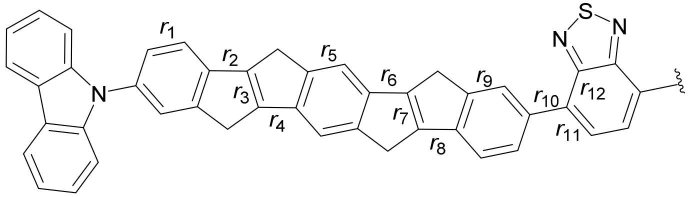

	bond length / Å											
	r_1	r_2	r_3	r_4	r_5	r_6	r_7	r_8	r_9	r_{10}	r_{11}	r_{12}
S_0	1.39133	1.45477	1.35049	1.45442	1.38341	1.45416	1.35103	1.45389	1.37850	1.47876	1.36967	1.43819
S_1	1.39066 (−0.05%)	1.45270 (−0.14%)	1.35292 (+0.18%)	1.44966 (−0.33%)	1.37992 (−0.25%)	1.44186 (−0.85%)	1.36385 (+0.95)	1.43407 (−1.36%)	1.37003 (−0.64%)	1.44721 (−2.13%)	1.41813 (+3.54%)	1.43819 (±0%)
S_3	1.38718 (−0.30%)	1.43835 (−1.13%)	1.36779 (+1.28%)	1.43279 (−1.48%)	1.37564 (−0.56%)	1.43513 (−1.31%)	1.36584 (+1.10%)	1.43944 (−0.99%)	1.37553 (−0.14%)	1.47214 (−0.45%)	1.38186 (+0.89%)	1.43560 (−0.18%)

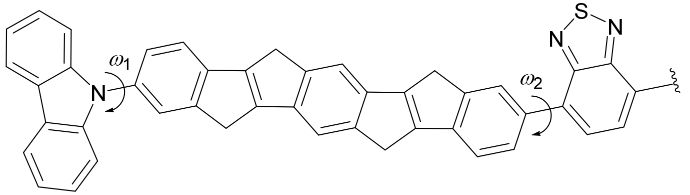

	dihedral angle / °	
	ω_1	ω_2
S_0	58.19	38.48
S_1	57.42	17.40
S_3	54.86	34.01

Appendix 1. NMR spectra

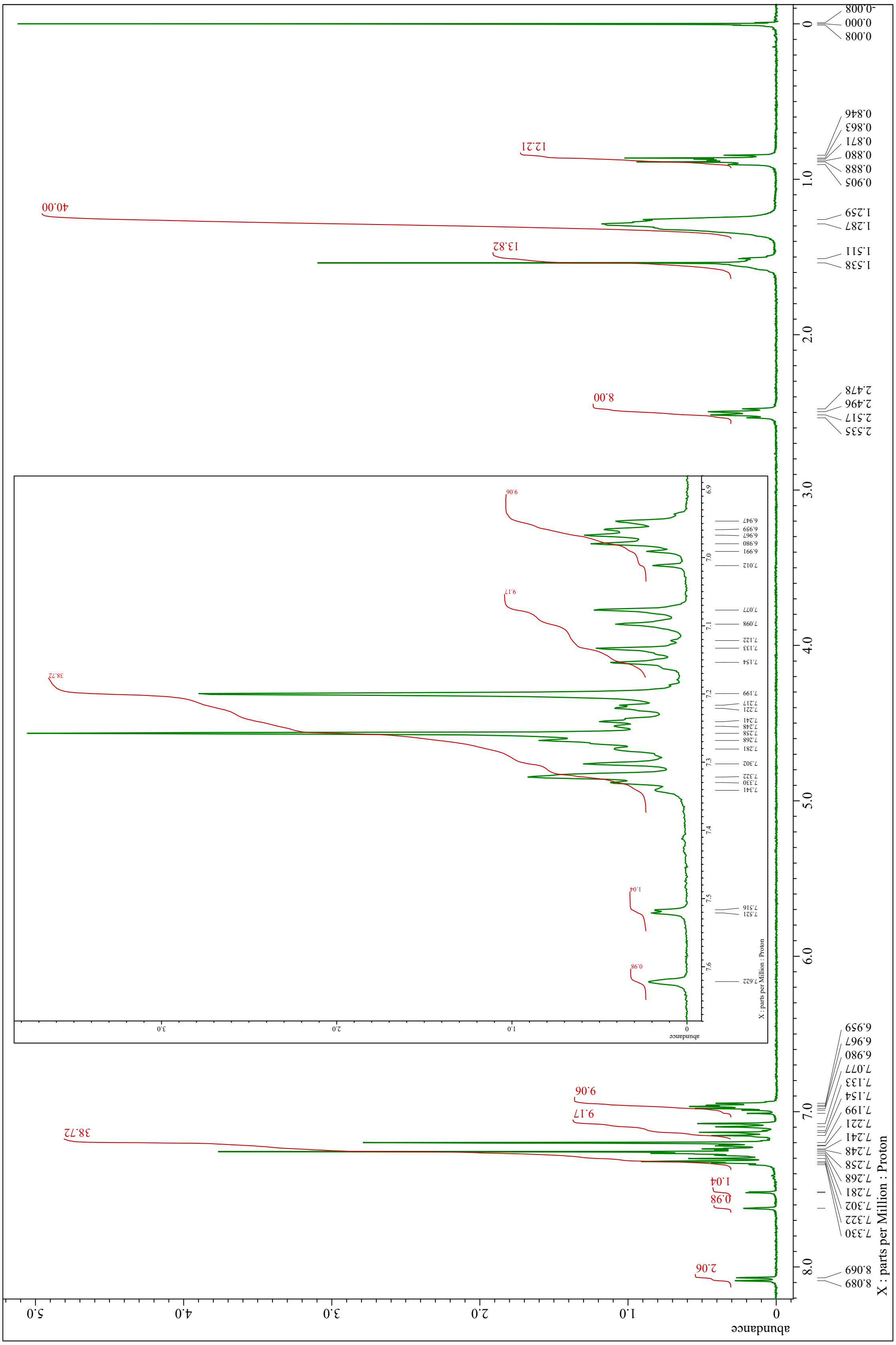

Figure SA1. ^{1}H NMR of **Cz-COPV2-Br**.

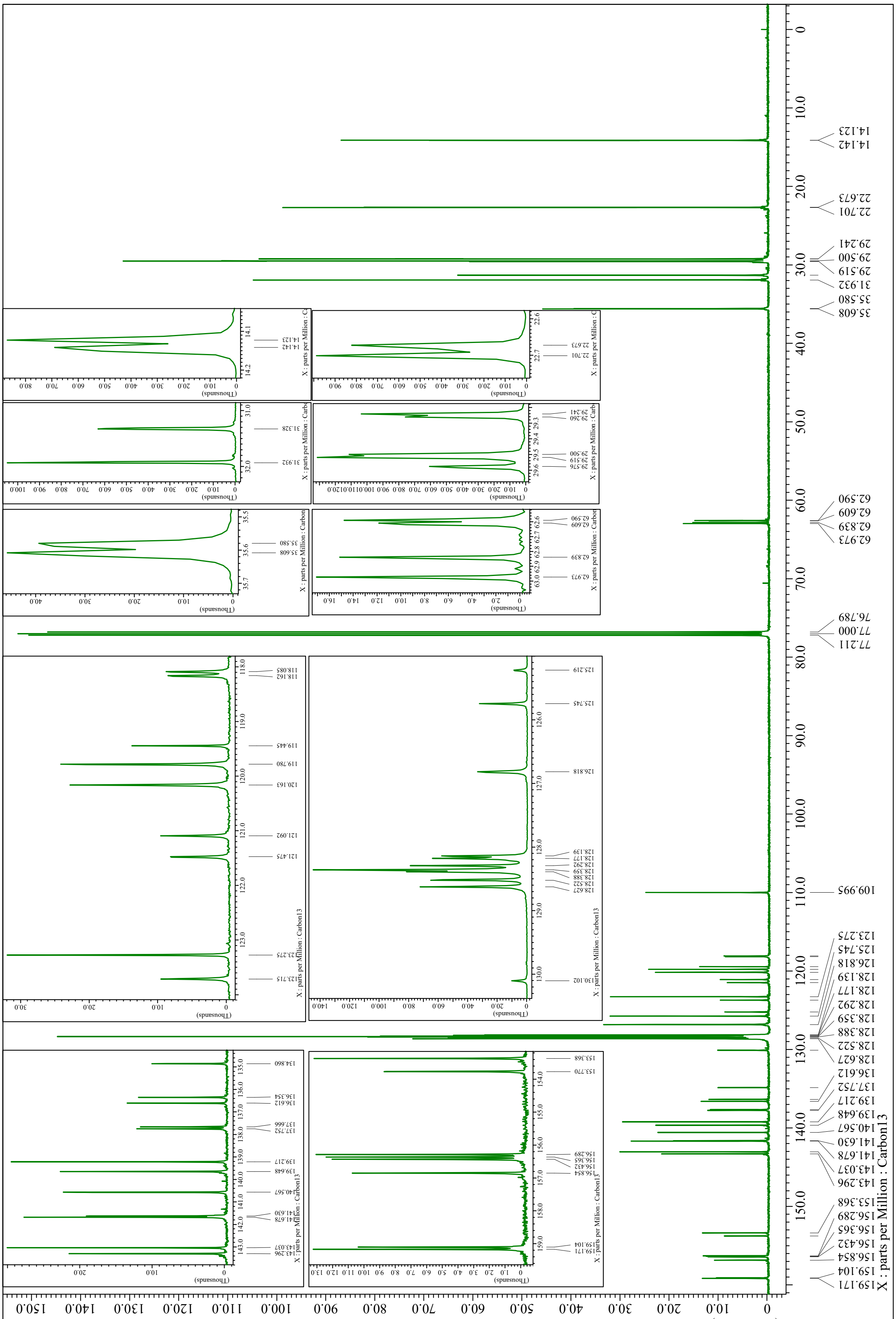

Figure SA2. ^{13}C NMR of **Cz-COPV2-Br**.

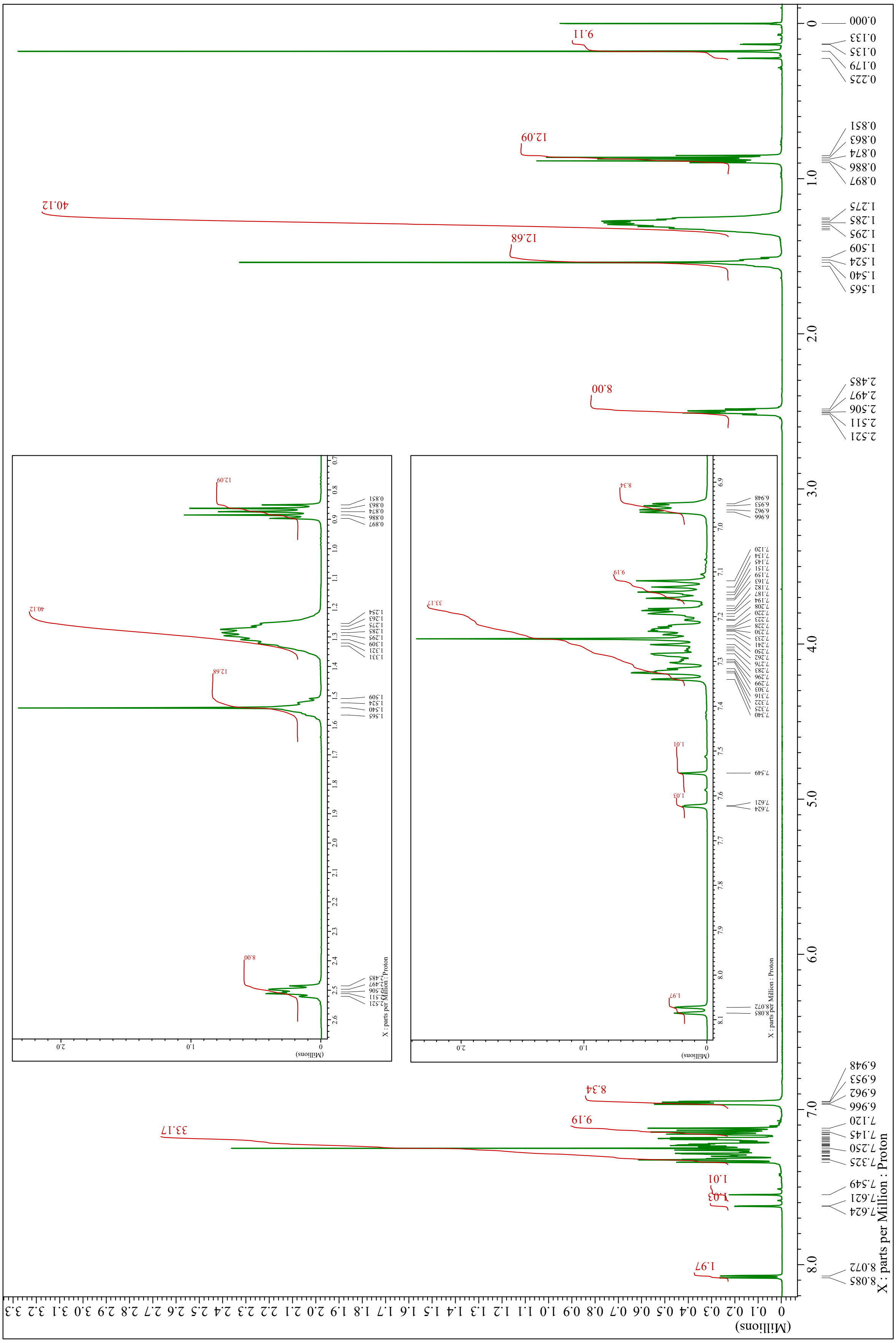

Figure SA3. ^{1}H NMR of **Cz-COPV2-SnMe$_3$**.

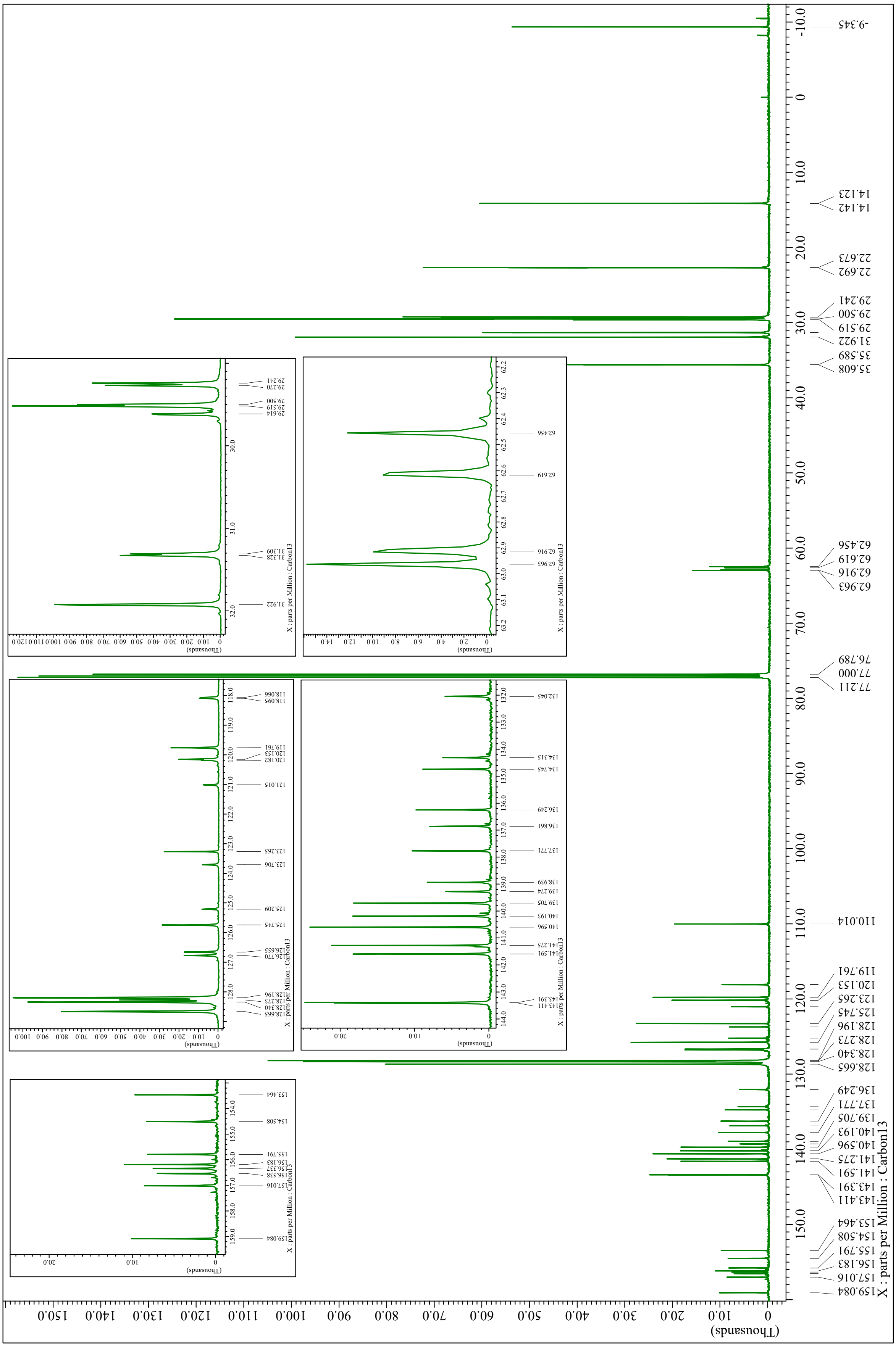

Figure SA4. ^{13}C NMR of **Cz-COPV2-SnMe$_3$**.

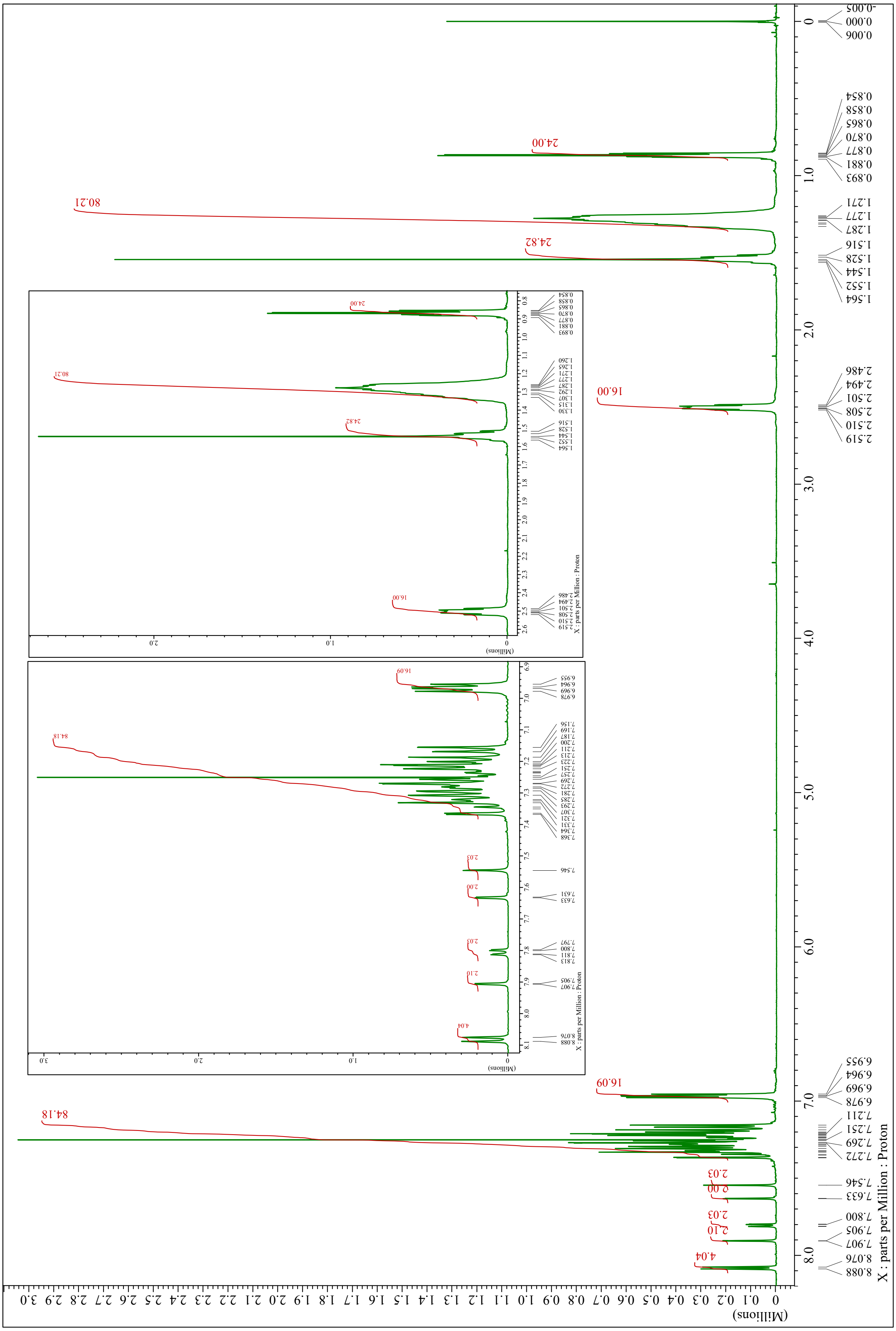

Figure SA5. ^{1}H NMR of **Cz-COPV2-BTz-COPV2-Cz**.

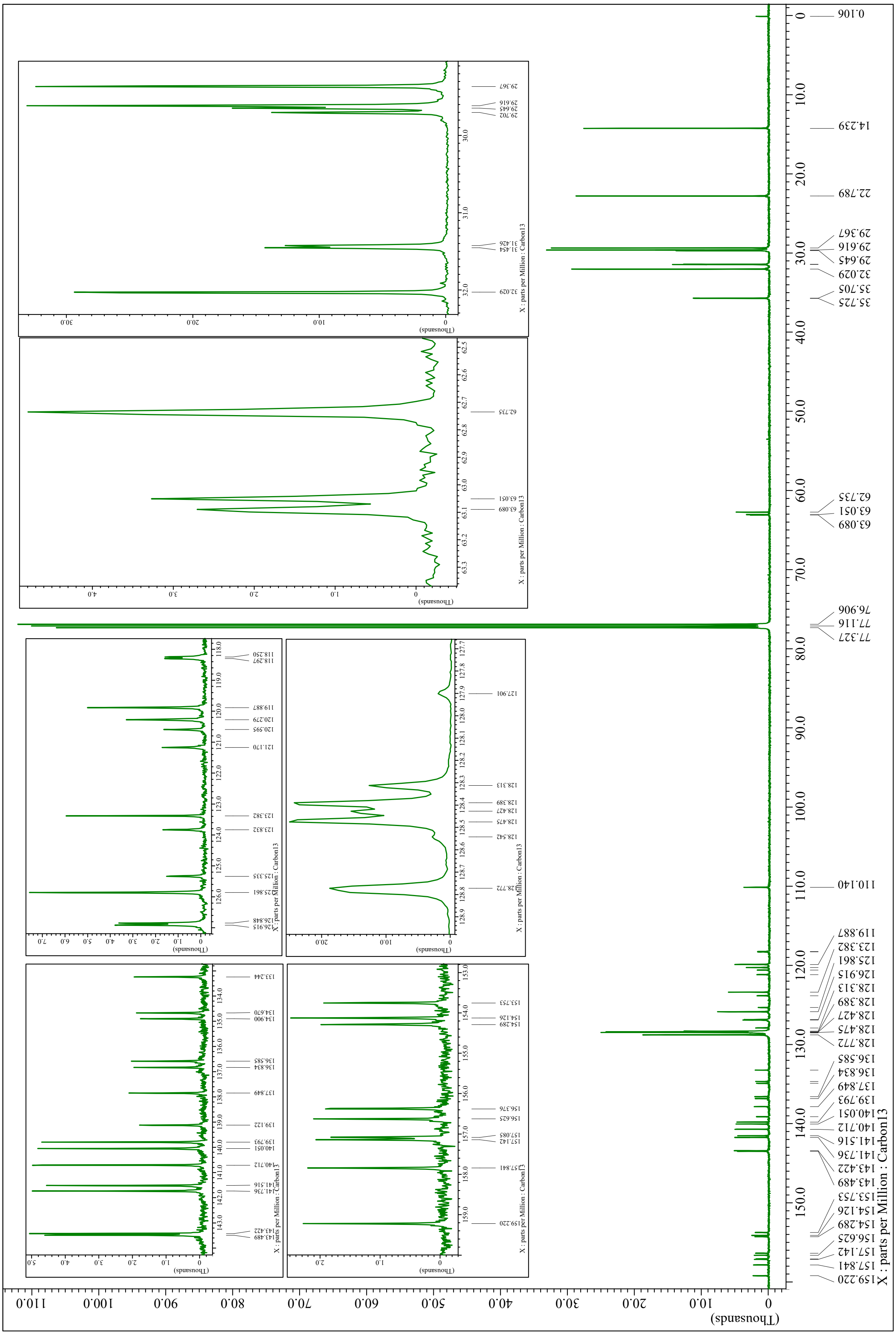

Figure SA6. ^{13}C NMR of **Cz-COPV2-BTz-COPV2-Cz**.

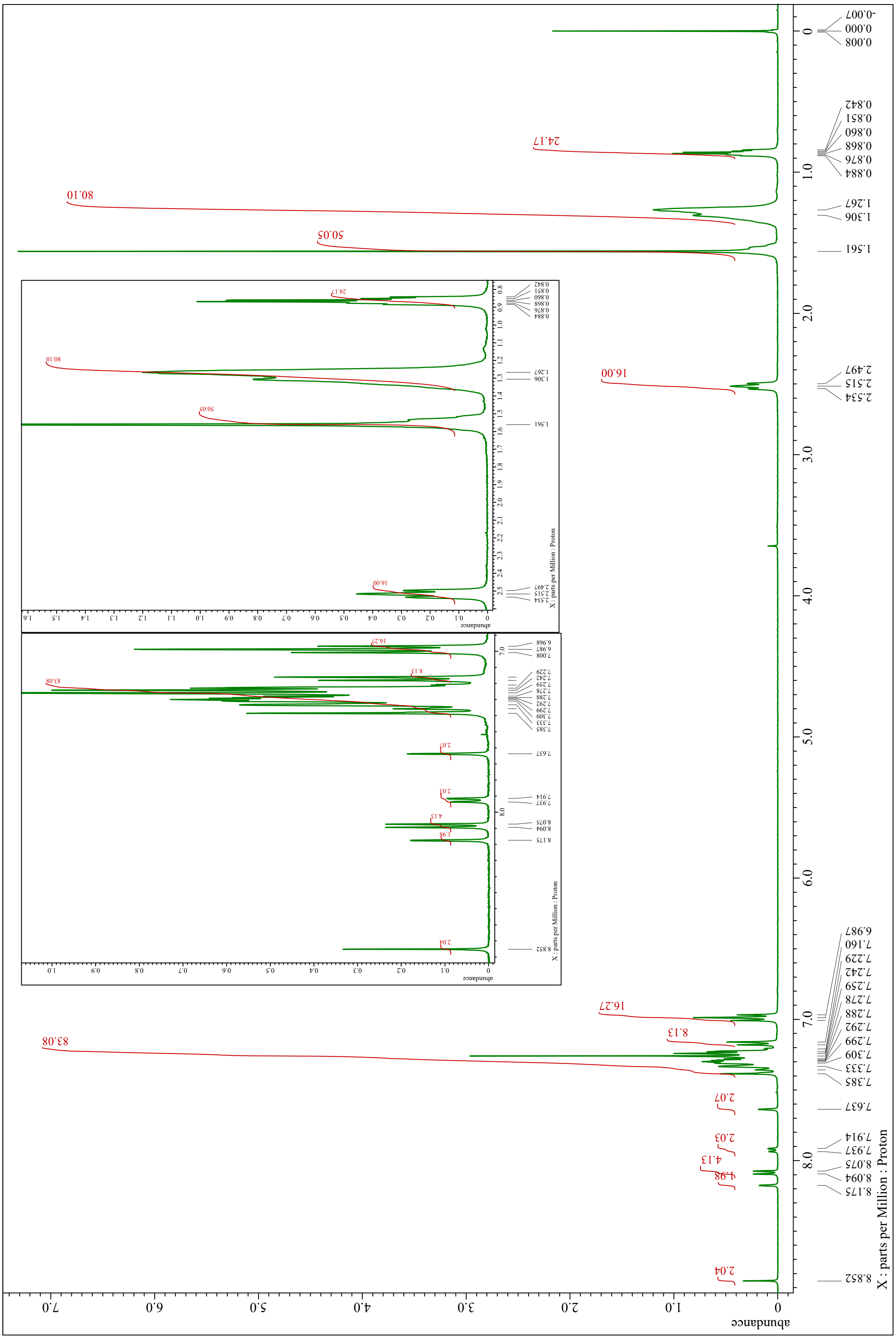

Figure SA7. ^{1}H NMR of **Cz-COPV2-NTz-COPV2-Cz.**

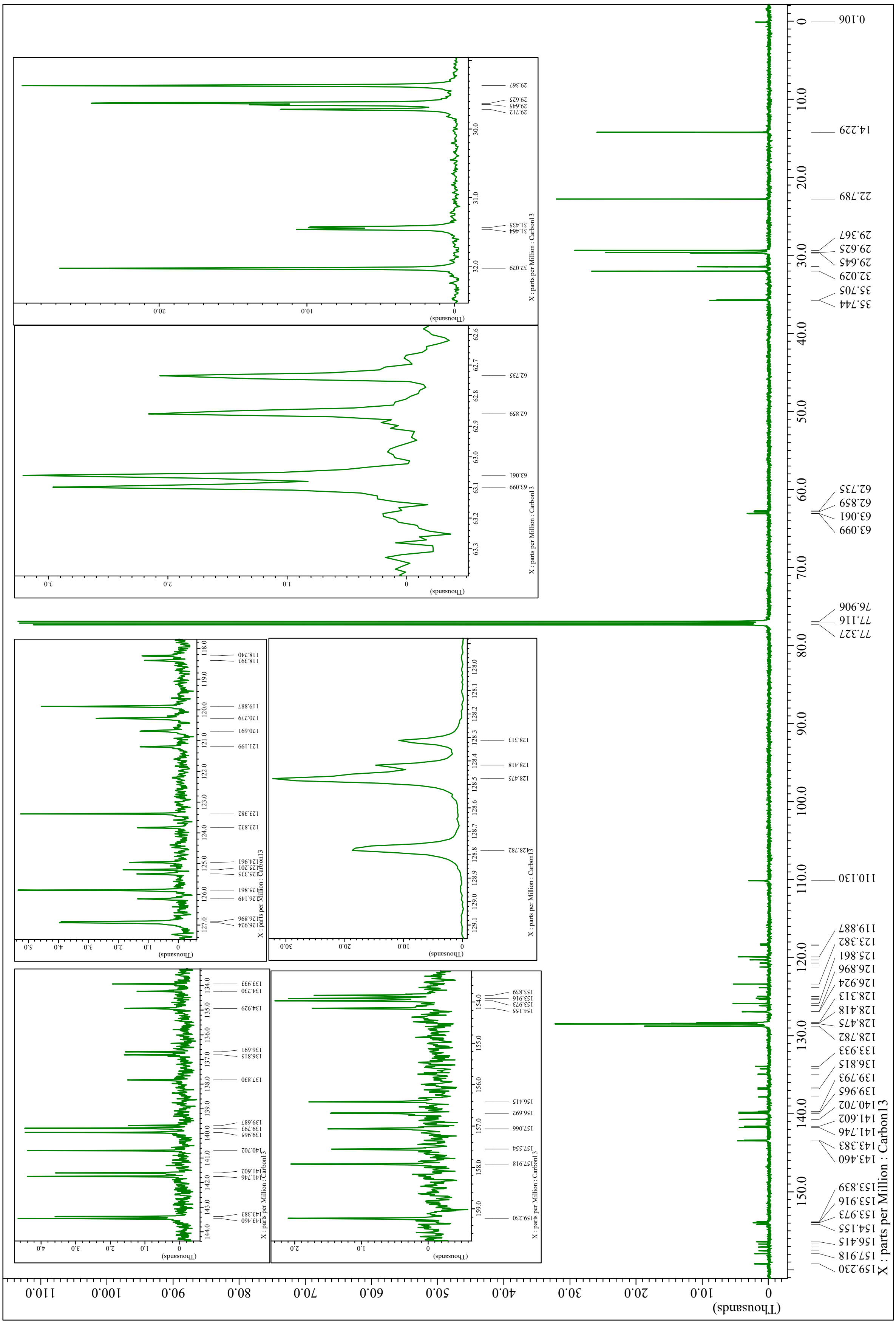

Figure SA8. ^{13}C NMR of **Cz-COPV2-NTz-COPV2-Cz**.

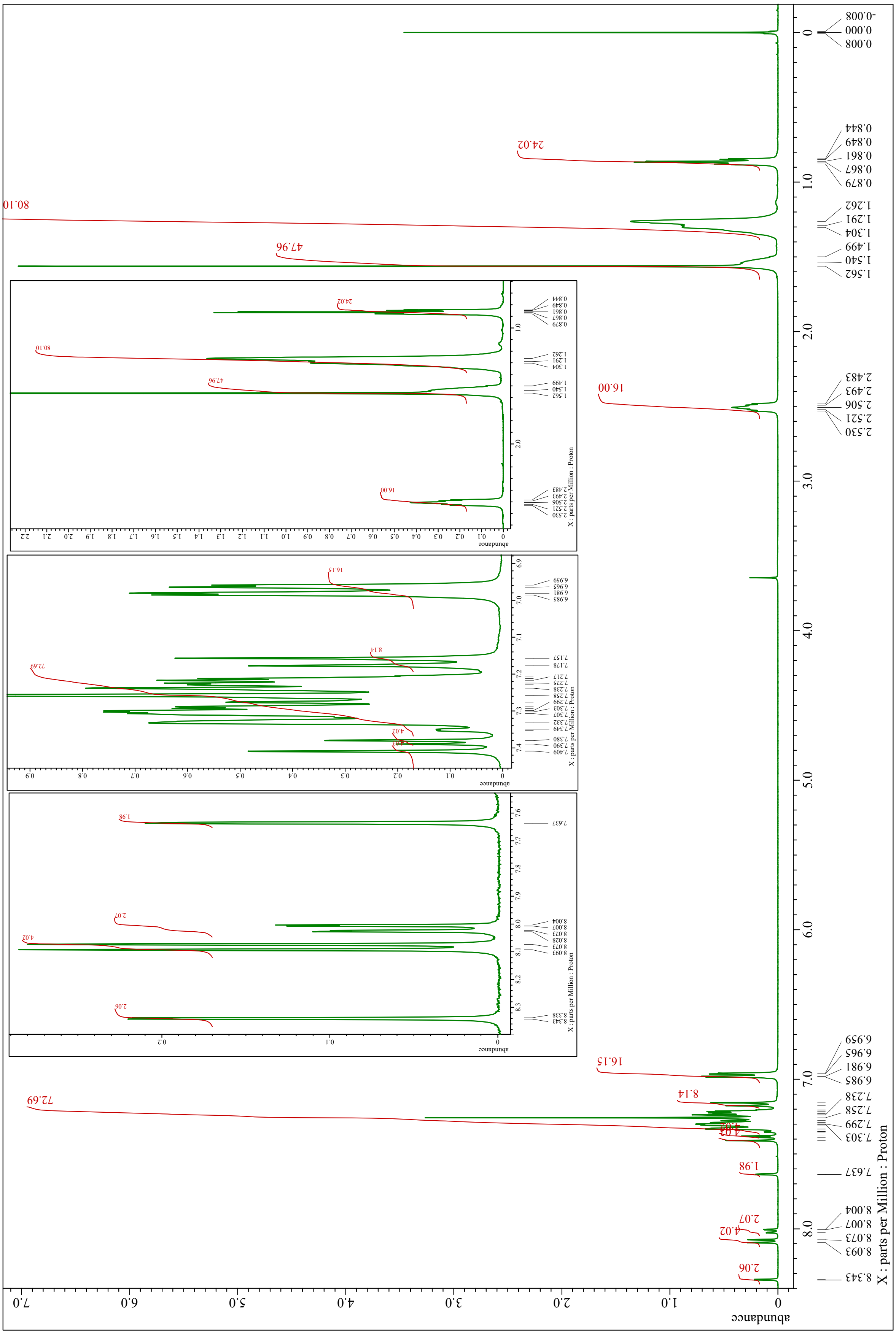

Figure SA9. ¹H NMR of **Cz-COPV2-BBTz-COPV2-Cz.**

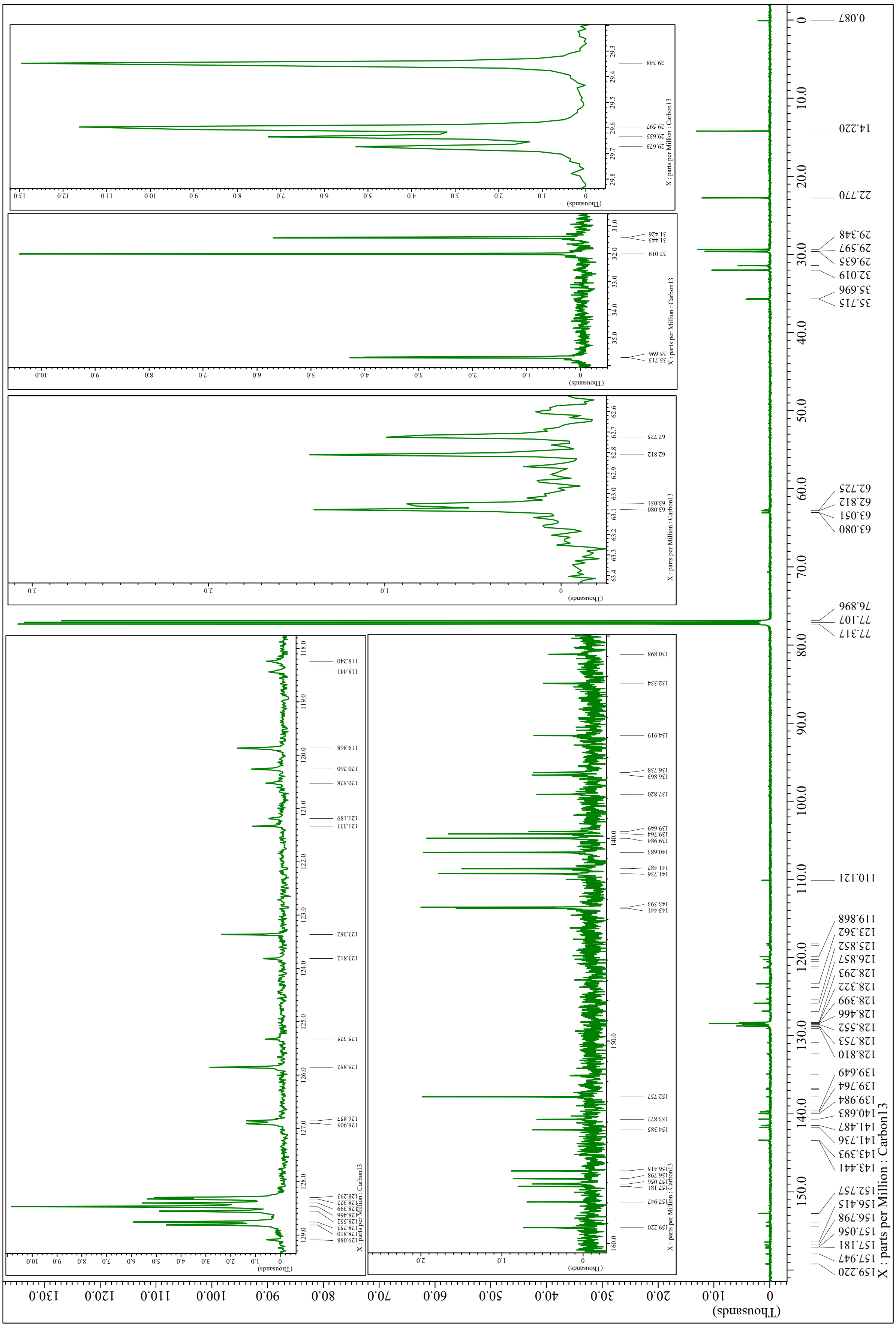

Figure SA10. ^{13}C NMR of **Cz-COPV2-BBTz-COPV2-Cz.**

Appendix 2. MS spectra

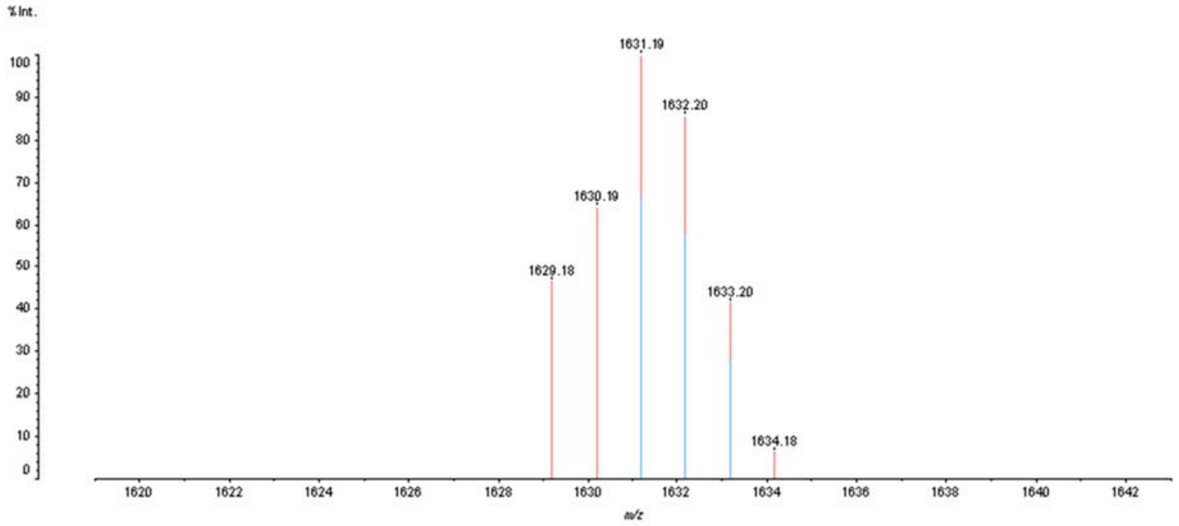

Figure SA11. MALDI-TOF MS of **Cz-COPV2-Br**.

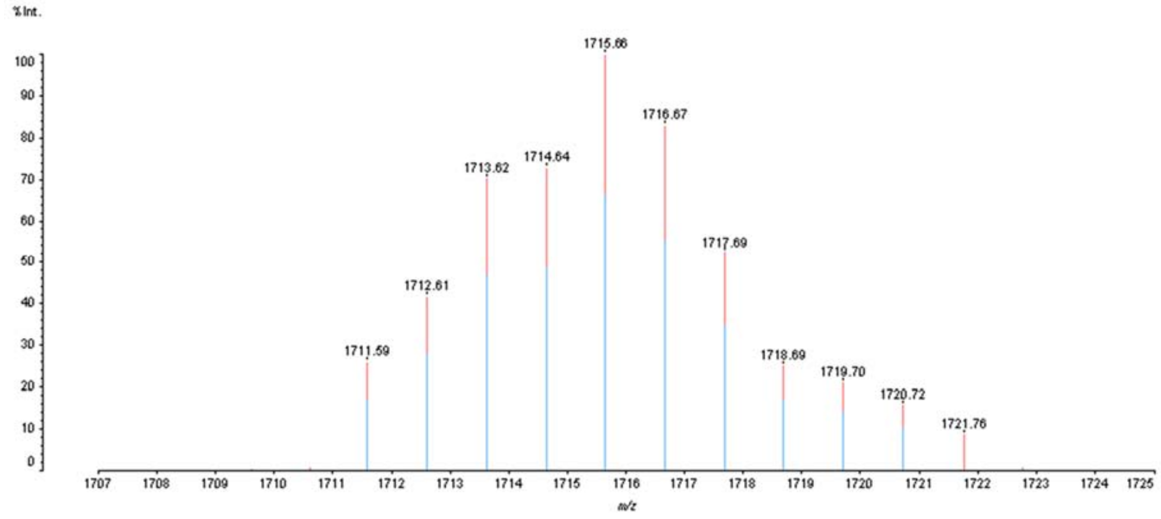

Figure SA12. MALDI-TOF MS of **Cz-COPV2-SnMe$_3$**.

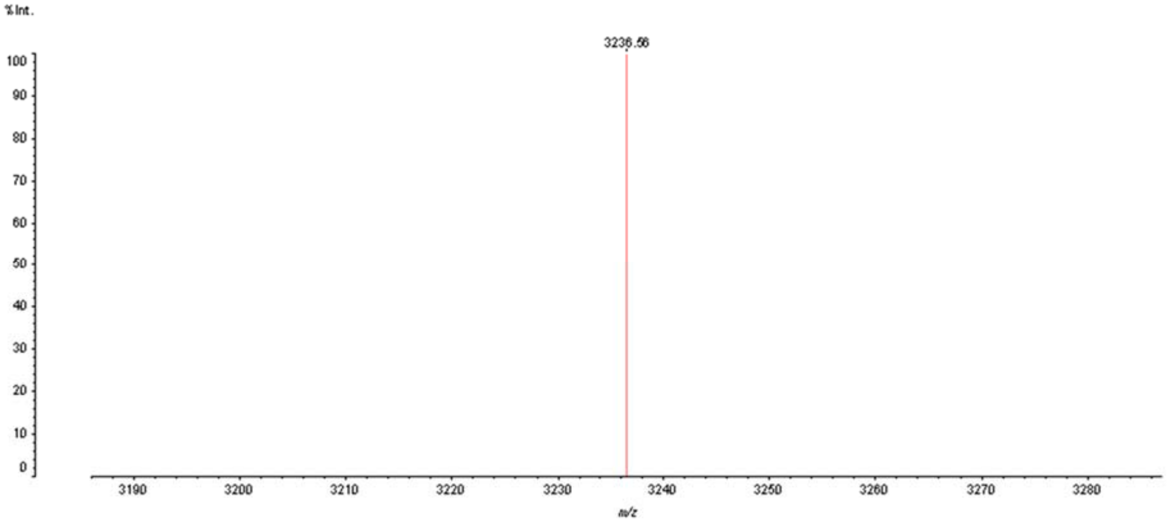

Figure SA13. MALDI-TOF MS of **Cz-COPV2-BTz-COPV2-Cz.**

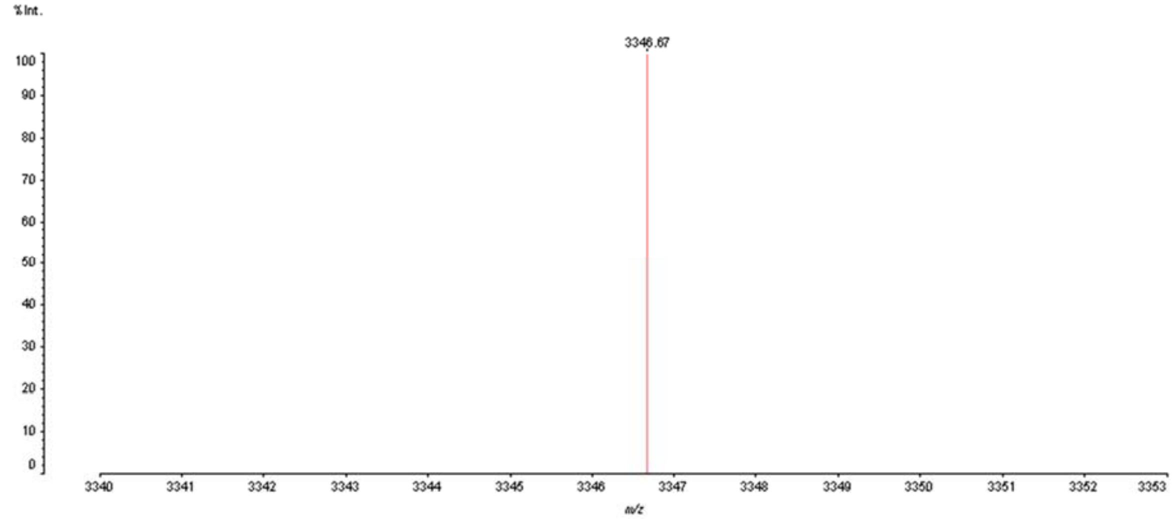

Figure SA14. MALDI-TOF MS of **Cz-COPV2-NTz-COPV2-Cz.**

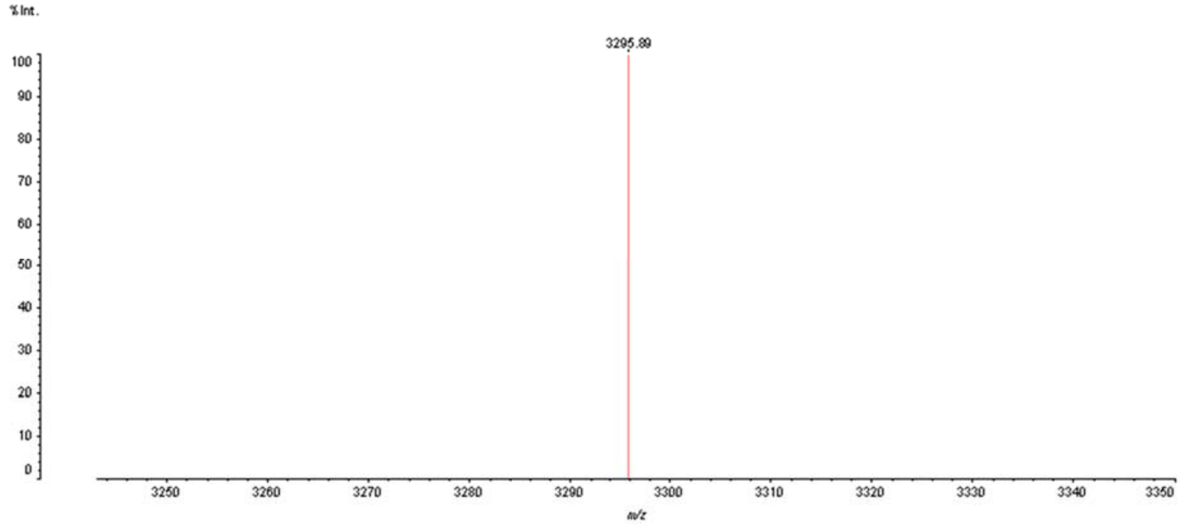

Figure SA15. MALDI-TOF MS of **Cz-COPV2-BBTz-COPV2-Cz.**

Appendix 3. Analytical GPC

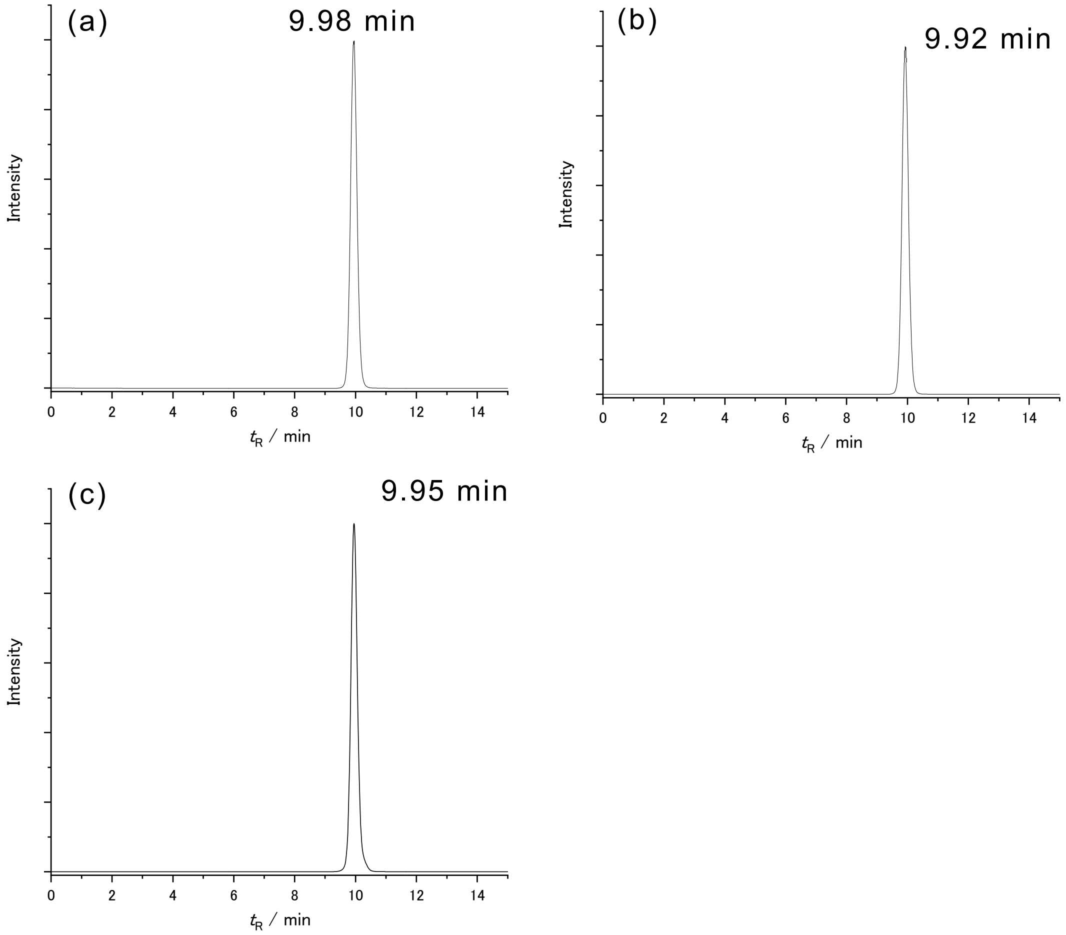

Figure SA16. Analytical GPC. (a) **Cz-COPV2-BTz-COPV2-Cz**, (b) **Cz-COPV2-NTz-COPV2-Cz**, (c) **Cz-COPV2-BBTz-COPV2-Cz**.

Appendix 4

(1) Optimized coordinates of a model compound: COPV2'-BTz

Ground state

Total energy = -1738.3797431 a.u.

Atomic Number	Coordinates (Angstroms) X	Y	Z
6	-8.093690	-0.819755	-0.067869
6	-10.102534	1.002222	0.522088
6	-7.771348	0.509882	0.281485
6	-9.412570	-1.228631	-0.119992
6	-10.420116	-0.308442	0.177664
6	-8.775918	1.423466	0.577165
1	-9.667541	-2.250411	-0.388120
1	-8.534609	2.447486	0.846561
1	-10.898158	1.704939	0.750617
6	-6.319991	0.621397	0.246323
6	-5.770678	-0.561572	-0.102290
6	-4.320441	-0.453409	-0.138134
6	-1.682245	0.370184	-0.031726
6	-3.323776	-1.382697	-0.437437
6	-3.995260	0.877855	0.211621
6	-2.679077	1.299675	0.267745
6	-2.007250	-0.960862	-0.381385
1	-3.576153	-2.405158	-0.705723
1	-2.426683	2.322060	0.536459
6	-5.266897	1.662762	0.483962
1	-5.366698	2.523791	-0.188670
1	-5.290082	2.059326	1.506676
6	-6.822629	-1.604942	-0.340253
1	-6.798741	-2.001623	-1.362909
1	-6.721983	-2.465628	0.332685
6	-0.232443	0.478200	-0.067437
6	0.317378	-0.705181	-0.417034
6	1.766533	-0.591970	-0.451355
6	4.436355	0.236832	-0.336176

6	2.089447	0.737654	-0.104676
6	2.779433	-1.500454	-0.733371
6	4.103317	-1.081627	-0.670156
6	3.404496	1.146422	-0.047066
1	2.548061	-2.527144	-1.000420
1	4.895398	-1.788302	-0.883350
1	3.651938	2.162607	0.246555
6	-0.736014	-1.746554	-0.653360
1	-0.636000	-2.606433	0.020616
1	-0.713206	-2.144658	-1.675430
6	0.818833	1.521829	0.172916
1	0.796504	1.912553	1.197814
1	0.715394	2.385808	-0.495262
16	8.310793	-1.837655	1.081766
7	9.152750	-0.502186	0.684788
7	6.813760	-1.364911	0.652003
6	8.275178	0.370056	0.195846
6	6.918784	-0.127718	0.173211
6	8.574969	1.681997	-0.267489
1	9.598086	2.038022	-0.246537
6	7.540031	2.437102	-0.723581
1	7.724869	3.441485	-1.091494
6	5.842549	0.696499	-0.314258
6	6.197782	1.947294	-0.747526
1	5.427872	2.596984	-1.150624
1	-11.459758	-0.618645	0.140012

1st excited state

Total energy = -1738.3686177 a.u.

Atomic Number	Coordinates (Angstroms) X	Y	Z
6	-8.068962	-0.867106	-0.028704
6	-10.105473	1.001013	0.251474
6	-7.766817	0.504070	0.135047
6	-9.382799	-1.293908	-0.051097

6	-10.402772	-0.350800	0.090231
6	-8.786330	1.441517	0.275501
1	-9.624415	-2.345663	-0.176466
1	-8.559156	2.495777	0.401548
1	-10.913095	1.718283	0.359672
6	-6.323194	0.628498	0.117085
6	-5.748796	-0.591590	-0.048125
6	-4.314312	-0.471646	-0.066131
6	-1.681604	0.390055	-0.019065
6	-3.300477	-1.429067	-0.208779
6	-4.003949	0.910205	0.099119
6	-2.702782	1.349201	0.124032
6	-1.995197	-0.993323	-0.184495
1	-3.542729	-2.480501	-0.334102
1	-2.461150	2.400733	0.249921
6	-5.289642	1.707707	0.228294
1	-5.382831	2.462936	-0.561828
1	-5.342963	2.244248	1.183614
6	-6.786773	-1.669820	-0.158659
1	-6.735427	-2.206440	-1.113990
1	-6.695047	-2.424688	0.631907
6	-0.264569	0.514058	-0.035333
6	0.326758	-0.723907	-0.202770
6	1.736723	-0.600885	-0.215386
6	4.424768	0.277186	-0.153676
6	2.053911	0.784249	-0.052630
6	2.780890	-1.535070	-0.336796
6	4.083964	-1.103527	-0.298854
6	3.349315	1.209038	-0.023516
1	2.562540	-2.591523	-0.458982
1	4.891435	-1.816942	-0.375332
1	3.562692	2.258437	0.139037
6	-0.714104	-1.797504	-0.313323
1	-0.622353	-2.552158	0.477177
1	-0.661113	-2.334518	-1.268133
6	0.772076	1.590973	0.079010
1	0.716639	2.124909	1.035824
1	0.678572	2.347973	-0.709451

16	8.453234	-1.965781	0.489509
7	9.219859	-0.511117	0.332971
7	6.884443	-1.459344	0.271275
6	8.269430	0.396841	0.102136
6	6.923015	-0.146554	0.067430
6	8.490699	1.774427	-0.097438
1	9.504468	2.157662	-0.073567
6	7.399425	2.598939	-0.328836
1	7.556694	3.658235	-0.506718
6	5.797619	0.732158	-0.143969
6	6.100827	2.100023	-0.345591
1	5.298844	2.790789	-0.573389
1	-11.438672	-0.674550	0.074270

----

(2) Optimized coordinates of a model compound: BTz-COPV2-BTz

Ground state

Total energy = -2475.7243864 a.u.

Atomic Number	Coordinates (Angstroms) X	Y	Z
6	7.438598	-0.342085	-0.034131
6	4.783088	0.537234	-0.013430
6	7.130344	1.023906	-0.040583
6	6.388602	-1.276435	-0.026905
6	5.080340	-0.842504	-0.016171
6	5.813747	1.469091	-0.026305
1	6.615320	-2.338406	-0.056445
1	5.601694	2.533967	-0.025675
6	3.334700	0.664752	-0.007962
6	2.761076	-0.558344	-0.011017
6	1.312076	-0.435870	-0.005819
6	-1.312076	0.435870	0.005819
6	0.296377	-1.392660	-0.005897
6	1.012970	0.946436	-0.000023
6	-0.296377	1.392660	0.005897
6	-1.012970	-0.946436	0.000023

1	0.529233	-2.454183	-0.010468
1	-0.529233	2.454183	0.010468
6	3.792849	-1.648044	-0.019872
1	3.717050	-2.291487	-0.905207
1	3.718017	-2.306317	0.854588
6	2.300978	1.751516	-0.001777
1	2.372269	2.404899	-0.880199
1	2.377469	2.400740	0.879292
6	-2.761076	0.558344	0.011017
6	-3.334700	-0.664752	0.007962
6	-4.783088	-0.537234	0.013430
6	-7.438598	0.342085	0.034131
6	-5.080340	0.842504	0.016171
6	-5.813747	-1.469091	0.026305
6	-7.130344	-1.023906	0.040583
6	-6.388602	1.276435	0.026905
1	-5.601694	-2.533967	0.025675
1	-7.936215	-1.746809	0.056957
1	-6.615320	2.338406	0.056445
6	-2.300978	-1.751516	0.001777
1	-2.377469	-2.400740	-0.879292
1	-2.372269	-2.404899	0.880199
6	-3.792849	1.648044	0.019872
1	-3.718017	2.306317	-0.854588
1	-3.717050	2.291487	0.905207
1	7.936215	1.746809	-0.056957
6	8.839409	-0.817425	-0.001633
6	11.560194	-1.831668	0.081552
6	9.209470	-1.916876	0.728118
6	9.893538	-0.170351	-0.739958
6	11.244231	-0.681925	-0.695696
6	10.545965	-2.421006	0.769495
1	8.457627	-2.423072	1.324586
1	10.743863	-3.296805	1.379489
1	12.578933	-2.199851	0.105832
6	-8.839409	0.817425	0.001633
6	-11.560194	1.831668	-0.081552
6	-9.893538	0.170351	0.739958

6	-9.209470	1.916876	-0.728118
6	-10.545965	2.421006	-0.769495
6	-11.244231	0.681925	0.695696
1	-8.457627	2.423072	-1.324586
1	-10.743863	3.296805	-1.379489
1	-12.578933	2.199851	-0.105832
7	9.770776	0.899465	-1.521872
7	12.100345	0.014684	-1.438550
7	-9.770776	-0.899465	1.521872
7	-12.100345	-0.014684	1.438550
16	-11.246561	-1.214722	2.131971
16	11.246561	1.214722	-2.131971

1st excited state

Total energy = -2475.7151918 a.u.

Atomic Number	Coordinates (Angstroms) X	Y	Z
6	7.413234	-0.345117	-0.074352
6	4.742083	0.546225	-0.080126
6	7.093213	1.030373	-0.167469
6	6.347851	-1.275562	0.005414
6	5.047826	-0.843567	0.001476
6	5.788661	1.477257	-0.163837
1	6.564886	-2.338365	0.030909
1	5.578329	2.540605	-0.224566
6	3.327605	0.678226	-0.070766
6	2.727773	-0.565615	0.007870
6	1.318680	-0.439645	0.013662
6	-1.318680	0.439645	-0.013662
6	0.287291	-1.403700	0.079608
6	1.007730	0.962020	-0.065686
6	-0.287291	1.403700	-0.079608
6	-1.007730	-0.962020	0.065686
1	0.525008	-2.461925	0.139779
1	-0.525008	2.461925	-0.139779

6	3.762220	-1.652061	0.058837
1	3.678313	-2.350806	-0.782848
1	3.694758	-2.253231	0.974075
6	2.296435	1.764796	-0.125846
1	2.364200	2.362664	-1.043451
1	2.380714	2.468169	0.712123
6	-2.727773	0.565615	-0.007870
6	-3.327605	-0.678226	0.070766
6	-4.742083	-0.546225	0.080126
6	-7.413234	0.345117	0.074352
6	-5.047826	0.843567	-0.001476
6	-5.788661	-1.477257	0.163837
6	-7.093213	-1.030373	0.167469
6	-6.347851	1.275562	-0.005414
1	-5.578329	-2.540605	0.224566
1	-7.899769	-1.746250	0.242224
1	-6.564886	2.338365	-0.030909
6	-2.296435	-1.764796	0.125846
1	-2.380714	-2.468169	-0.712123
1	-2.364200	-2.362664	1.043451
6	-3.762220	1.652061	-0.058837
1	-3.694758	2.253231	-0.974075
1	-3.678313	2.350806	0.782848
1	7.899769	1.746250	-0.242224
6	8.797585	-0.815930	-0.033797
6	11.511271	-1.855337	0.065695
6	9.137529	-2.007002	0.589875
6	9.892903	-0.094509	-0.639667
6	11.240403	-0.626918	-0.589552
6	10.457769	-2.513544	0.639668
1	8.364682	-2.569231	1.101598
1	10.628688	-3.450639	1.160550
1	12.525858	-2.234069	0.097862
6	-8.797585	0.815930	0.033797
6	-11.511271	1.855337	-0.065695
6	-9.892903	0.094509	0.639667
6	-9.137529	2.007002	-0.589875
6	-10.457769	2.513544	-0.639668

6	-11.240403	0.626918	0.589552
1	-8.364682	2.569231	-1.101598
1	-10.628688	3.450639	-1.160550
1	-12.525858	2.234069	-0.097862
7	9.817801	1.054584	-1.302779
7	12.138508	0.136742	-1.207716
7	-9.817801	-1.054584	1.302779
7	-12.138508	-0.136742	1.207716
16	-11.330832	-1.424083	1.805545
16	11.330832	1.424083	-1.805545

----

(3) Optimized coordinates of a model compound: COPV2'-BTz-COPV2'

Ground state

Total energy = -2738.2322124 a.u.

Atomic Number	Coordinates (Angstroms) X	Y	Z
6	1.745000	-0.201376	2.947396
6	1.614322	-0.308432	5.740971
6	2.886435	-0.465998	3.714648
6	0.522687	0.024021	3.604191
6	0.458689	-0.030116	4.979905
6	2.829348	-0.524335	5.102179
1	-0.362331	0.264272	3.021863
1	3.728096	-0.737806	5.672889
6	1.221161	-0.292624	7.140738
6	-0.096984	-0.017682	7.249557
6	-0.496258	-0.005595	8.647802
6	-0.627292	-0.127196	11.407864
6	-1.727746	0.221951	9.263348
6	0.659298	-0.290720	9.411394
6	0.604207	-0.353675	10.792428
6	-1.782746	0.158719	10.644082
1	-2.614355	0.441056	8.674186
1	1.490738	-0.573024	11.381560
6	-0.728149	0.183971	5.903033

1	-1.159564	1.185984	5.786866
1	-1.536298	-0.531156	5.705259
6	1.848526	-0.493687	8.488363
1	2.649932	0.227619	8.691169
1	2.288517	-1.492099	8.603354
6	-1.028009	-0.120034	12.806318
6	-2.345913	0.152258	12.914372
6	-2.744258	0.157106	14.314821
6	-2.886481	0.028696	17.082391
6	-1.589739	-0.129787	15.075523
6	-3.964130	0.378424	14.942623
6	-4.024571	0.311579	16.332660
6	-1.659237	-0.194023	16.454066
1	-4.855698	0.599351	14.363203
1	-4.970895	0.482165	16.837081
1	-0.774649	-0.414457	17.045562
6	-2.972177	0.359511	11.567253
1	-3.410445	1.359165	11.455938
1	-3.774687	-0.359600	11.360795
6	-0.399787	-0.328644	14.153089
1	0.402172	0.390994	14.359838
1	0.039414	-1.328078	14.262644
1	3.832363	-0.629508	3.213983
6	1.790155	-0.192673	1.469541
6	1.790155	-0.192673	-1.469541
6	0.759569	-0.682609	0.711629
6	2.901862	0.334123	0.724505
6	2.901862	0.334123	-0.724505
6	0.759569	-0.682609	-0.711629
1	-0.094169	-1.125242	1.214374
1	-0.094169	-1.125242	-1.214374
7	3.997322	0.881704	1.243795
7	3.997322	0.881704	-1.243795
16	4.935836	1.347607	0.000000
1	-2.952761	-0.019337	18.164892
6	1.745000	-0.201376	-2.947396
6	1.614322	-0.308432	-5.740971
6	0.522687	0.024021	-3.604191

6	2.886435	-0.465998	-3.714648
6	2.829348	-0.524335	-5.102179
6	0.458689	-0.030116	-4.979905
1	-0.362331	0.264272	-3.021863
1	3.832363	-0.629508	-3.213983
1	3.728096	-0.737806	-5.672889
6	1.221161	-0.292624	-7.140738
6	-0.096984	-0.017682	-7.249557
6	-0.496258	-0.005595	-8.647802
6	-0.627292	-0.127196	-11.407864
6	0.659298	-0.290720	-9.411394
6	-1.727746	0.221951	-9.263348
6	-1.782746	0.158719	-10.644082
6	0.604207	-0.353675	-10.792428
1	-2.614355	0.441056	-8.674186
1	1.490738	-0.573024	-11.381560
6	-1.028009	-0.120034	-12.806318
6	-2.345913	0.152258	-12.914372
6	-2.744258	0.157106	-14.314821
6	-2.886481	0.028696	-17.082391
6	-3.964130	0.378424	-14.942623
6	-1.589739	-0.129787	-15.075523
6	-1.659237	-0.194023	-16.454066
6	-4.024571	0.311579	-16.332660
1	-4.855698	0.599351	-14.363203
1	-0.774649	-0.414457	-17.045562
1	-4.970895	0.482165	-16.837081
1	-2.952761	-0.019337	-18.164892
6	-0.728149	0.183971	-5.903033
1	-1.159564	1.185984	-5.786866
1	-1.536298	-0.531156	-5.705259
6	-2.972177	0.359511	-11.567253
1	-3.410445	1.359165	-11.455938
1	-3.774687	-0.359600	-11.360795
6	-0.399787	-0.328644	-14.153089
1	0.039414	-1.328078	-14.262644
1	0.402172	0.390994	-14.359838
6	1.848526	-0.493687	-8.488363

1	2.288517	-1.492099	-8.603354
1	2.649932	0.227619	-8.691169

1st excited state

Total energy = - 2738.2195772 a.u.

Atomic Number	Coordinates (Angstroms) X	Y	Z
6	1.874794	-0.174843	2.914392
6	1.685195	-0.173276	5.732025
6	3.028335	-0.216083	3.743324
6	0.605491	-0.121217	3.559756
6	0.516967	-0.118213	4.924713
6	2.940738	-0.223260	5.120464
1	-0.299196	-0.039457	2.968379
1	3.845919	-0.266208	5.718721
6	1.263362	-0.149316	7.103110
6	-0.095812	-0.077417	7.178316
6	-0.521978	-0.049043	8.555708
6	-0.704550	-0.023185	11.316760
6	-1.793345	0.021520	9.137603
6	0.646092	-0.106417	9.358579
6	0.564771	-0.094047	10.737435
6	-1.872732	0.033806	10.515166
1	-2.686511	0.065677	8.520304
1	1.457903	-0.137205	11.354792
6	-0.714558	-0.047400	5.812423
1	-1.295447	0.866345	5.635997
1	-1.394505	-0.891010	5.641090
6	1.876611	-0.174725	8.471693
1	2.551784	0.672242	8.644239
1	2.461287	-1.084872	8.652866
6	-1.135322	0.009388	12.701064
6	-2.483925	0.081913	12.771692
6	-2.912415	0.115494	14.160919
6	-3.102482	0.147611	16.927583

6	-1.746630	0.059457	14.956346
6	-4.167913	0.187336	14.753891
6	-4.251834	0.202881	16.143678
6	-1.840076	0.075381	16.334824
1	-5.067754	0.230748	14.147514
1	-5.225112	0.258845	16.621672
1	-0.947617	0.032616	16.953238
6	-3.101248	0.104973	11.405370
1	-3.685098	1.015584	11.222412
1	-3.777927	-0.741501	11.234663
6	-0.517038	-0.012914	14.068212
1	0.159748	0.833645	14.238028
1	0.066578	-0.923572	14.251688
1	3.997701	-0.241250	3.267730
6	1.959602	-0.195012	1.470561
6	1.959602	-0.195012	-1.470561
6	0.826779	-0.534172	0.686947
6	3.152560	0.111635	0.728339
6	3.152560	0.111635	-0.728339
6	0.826779	-0.534172	-0.686947
1	-0.074727	-0.865611	1.186776
1	-0.074727	-0.865611	-1.186776
7	4.325695	0.450653	1.253873
7	4.325695	0.450653	-1.253873
16	5.368057	0.741811	0.000000
1	-3.187970	0.160790	18.009674
6	1.874794	-0.174843	-2.914392
6	1.685195	-0.173276	-5.732025
6	0.605491	-0.121217	-3.559756
6	3.028335	-0.216083	-3.743324
6	2.940738	-0.223260	-5.120464
6	0.516967	-0.118213	-4.924713
1	-0.299196	-0.039457	-2.968379
1	3.997701	-0.241250	-3.267730
1	3.845919	-0.266208	-5.718721
6	1.263362	-0.149316	-7.103110
6	-0.095812	-0.077417	-7.178316
6	-0.521978	-0.049043	-8.555708

6	-0.704550	-0.023185	-11.316760
6	0.646092	-0.106417	-9.358579
6	-1.793345	0.021520	-9.137603
6	-1.872732	0.033806	-10.515166
6	0.564771	-0.094047	-10.737435
1	-2.686511	0.065677	-8.520304
1	1.457903	-0.137205	-11.354792
6	-1.135322	0.009388	-12.701064
6	-2.483925	0.081913	-12.771692
6	-2.912415	0.115494	-14.160919
6	-3.102482	0.147611	-16.927583
6	-4.167913	0.187336	-14.753891
6	-1.746630	0.059457	-14.956346
6	-1.840076	0.075381	-16.334824
6	-4.251834	0.202881	-16.143678
1	-5.067754	0.230748	-14.147514
1	-0.947617	0.032616	-16.953238
1	-5.225112	0.258845	-16.621672
1	-3.187970	0.160790	-18.009674
6	-0.714558	-0.047400	-5.812423
1	-1.295447	0.866345	-5.635997
1	-1.394505	-0.891010	-5.641090
6	-3.101248	0.104973	-11.405370
1	-3.685098	1.015584	-11.222412
1	-3.777927	-0.741501	-11.234663
6	-0.517038	-0.012914	-14.068212
1	0.066578	-0.923572	-14.251688
1	0.159748	0.833645	-14.238028
6	1.876611	-0.174725	-8.471693
1	2.461287	-1.084872	-8.652866
1	2.551784	0.672242	-8.644239

----

(4) Optimized coordinates of a model compound: Cz-COPV2'-BTz-COPV2'-Cz

Ground state

Total energy = -3770.2069156 a.u.

Atomic Coordinates (Angstroms)

Number	X	Y	Z
6	-0.070896	-0.721009	0.189388
6	0.068918	0.708440	2.560736
6	-0.149234	-1.461758	1.419655
6	0.070896	0.721009	0.189388
6	0.149234	1.461758	1.419655
6	-0.068918	-0.708440	2.560736
1	-0.099181	-1.211170	3.521878
1	0.099181	1.211170	3.521878
7	0.125578	1.237312	-1.035396
7	-0.125578	-1.237312	-1.035396
16	-0.000000	0.000000	-2.083487
6	-0.320654	-2.929986	1.460249
6	-0.630768	-5.705309	1.646060
6	0.326090	-3.769407	0.537259
6	-1.120731	-3.507176	2.455014
6	-1.281406	-4.883705	2.558246
6	0.172684	-5.135885	0.634570
1	0.938911	-3.336708	-0.244496
1	-1.649731	-2.859583	3.146998
1	-1.915324	-5.301857	3.334339
6	-0.586436	-7.147824	1.470078
6	0.197449	-7.462261	0.415597
6	0.244131	-8.904406	0.234994
6	-0.063314	-11.646921	0.408621
6	0.903363	-9.705037	-0.698393
6	-0.562564	-9.475794	1.246536
6	-0.722567	-10.846448	1.342185
6	0.742771	-11.075785	-0.603092
1	1.522120	-9.263119	-1.474817
1	-1.341929	-11.288119	2.118341
6	-0.017027	-13.089110	0.226190
6	0.764459	-13.403248	-0.829473
6	0.804561	-14.846238	-1.009795
6	0.487115	-17.601815	-0.843813
6	1.445309	-15.663306	-1.933394
6	-0.000000	-15.414865	0.002236

6	-0.168564	-16.782033	0.082662
6	1.290185	-17.042647	-1.837670
1	2.069992	-15.239882	-2.713820
1	-0.802334	-17.233141	0.840104
1	1.799582	-17.701682	-2.532768
6	-0.588202	-14.317703	0.871202
1	-1.685022	-14.330447	0.857809
1	-0.285649	-14.423386	1.920326
6	1.332686	-12.172975	-1.472374
1	2.429614	-12.158804	-1.458419
1	1.030672	-12.068030	-2.521848
6	-1.154367	-8.378039	2.113578
1	-2.251254	-8.393221	2.097761
1	-0.854111	-8.481512	3.163714
6	0.765924	-6.233510	-0.231041
1	1.862782	-6.218663	-0.216416
1	0.464242	-6.130870	-1.280705
7	0.330859	-19.009727	-0.770761
6	0.637424	-19.807407	0.329167
6	1.104481	-21.786454	2.203560
6	1.178503	-19.442702	1.560360
6	0.340966	-21.150707	0.022058
6	0.576560	-22.142901	0.972905
6	1.403961	-20.447933	2.488413
1	1.420443	-18.409428	1.782458
1	0.352125	-23.181706	0.749113
1	1.824685	-20.189788	3.455456
1	1.291865	-22.548831	2.952794
6	-0.161322	-19.830081	-1.783516
6	-1.084382	-21.841049	-3.442257
6	-0.171720	-21.165062	-1.331489
6	-0.620237	-19.485950	-3.053403
6	-1.077602	-20.507563	-3.871696
6	-0.635748	-22.174260	-2.174304
1	-0.622966	-18.454698	-3.388118
1	-1.439793	-20.265879	-4.866284
1	-0.647985	-23.207083	-1.838629
1	-1.447218	-22.616921	-4.108636

6	0.320654	2.929986	1.460249
6	0.630768	5.705309	1.646060
6	-0.326090	3.769407	0.537259
6	1.120731	3.507176	2.455014
6	1.281406	4.883705	2.558246
6	-0.172684	5.135885	0.634570
1	-0.938911	3.336708	-0.244496
1	1.649731	2.859583	3.146998
1	1.915324	5.301857	3.334339
6	0.586436	7.147824	1.470078
6	-0.197449	7.462261	0.415597
6	-0.244131	8.904406	0.234994
6	0.063314	11.646921	0.408621
6	-0.903363	9.705037	-0.698393
6	0.562564	9.475794	1.246536
6	0.722567	10.846448	1.342185
6	-0.742771	11.075785	-0.603092
1	-1.522120	9.263119	-1.474817
1	1.341929	11.288119	2.118341
6	0.017027	13.089110	0.226190
6	-0.764459	13.403248	-0.829473
6	-0.804561	14.846238	-1.009795
6	-0.487115	17.601815	-0.843813
6	-1.445309	15.663306	-1.933394
6	-0.000000	15.414865	0.002236
6	0.168564	16.782033	0.082662
6	-1.290185	17.042647	-1.837670
1	-2.069992	15.239882	-2.713820
1	0.802334	17.233141	0.840104
1	-1.799582	17.701682	-2.532768
6	0.588202	14.317703	0.871202
1	1.685022	14.330447	0.857809
1	0.285649	14.423386	1.920326
6	-1.332686	12.172975	-1.472374
1	-2.429614	12.158804	-1.458419
1	-1.030672	12.068030	-2.521848
6	1.154367	8.378039	2.113578
1	2.251254	8.393221	2.097761

1	0.854111	8.481512	3.163714
6	-0.765924	6.233510	-0.231041
1	-1.862782	6.218663	-0.216416
1	-0.464242	6.130870	-1.280705
7	-0.330859	19.009727	-0.770761
6	-0.637424	19.807407	0.329167
6	-1.104481	21.786454	2.203560
6	-1.178503	19.442702	1.560360
6	-0.340966	21.150707	0.022058
6	-0.576560	22.142901	0.972905
6	-1.403961	20.447933	2.488413
1	-1.420443	18.409428	1.782458
1	-0.352125	23.181706	0.749113
1	-1.824685	20.189788	3.455456
1	-1.291865	22.548831	2.952794
6	0.161322	19.830081	-1.783516
6	1.084382	21.841049	-3.442257
6	0.171720	21.165062	-1.331489
6	0.620237	19.485950	-3.053403
6	1.077602	20.507563	-3.871696
6	0.635748	22.174260	-2.174304
1	0.622966	18.454698	-3.388118
1	1.439793	20.265879	-4.866284
1	0.647985	23.207083	-1.838629
1	1.447218	22.616921	-4.108636

1st excited state

Total energy = -3770.1938661 a.u.

Atomic Number	Coordinates (Angstroms) X	Y	Z
6	-0.026635	-0.728032	0.362675
6	0.045159	0.685536	2.775159
6	-0.082797	-1.469258	1.593867
6	0.026635	0.728032	0.362675
6	0.082797	1.469258	1.593867

6	-0.045159	-0.685536	2.775159
1	-0.047405	-1.183072	3.736335
1	0.047405	1.183072	3.736335
7	0.043769	1.252805	-0.859058
7	-0.043769	-1.252805	-0.859058
16	-0.000000	0.000000	-1.941074
6	-0.175641	-2.911685	1.665963
6	-0.335182	-5.722954	1.902132
6	0.139019	-3.748952	0.559975
6	-0.583550	-3.548438	2.872031
6	-0.667728	-4.917572	2.997135
6	0.066242	-5.111246	0.685846
1	0.432189	-3.290261	-0.373872
1	-0.880688	-2.941814	3.718096
1	-0.998499	-5.359933	3.931977
6	-0.310780	-7.142986	1.703494
6	0.085478	-7.432914	0.431094
6	0.110982	-8.860086	0.227437
6	-0.034463	-11.613828	0.450015
6	0.442840	-9.635702	-0.889703
6	-0.291303	-9.467306	1.444958
6	-0.366355	-10.841014	1.565775
6	0.366355	-11.008078	-0.767630
1	0.749589	-9.167447	-1.820899
1	-0.673713	-11.308985	2.497028
6	-0.007257	-13.048092	0.241056
6	0.382490	-13.331783	-1.023073
6	0.404296	-14.769751	-1.228300
6	0.247652	-17.535311	-1.008624
6	0.720173	-15.560159	-2.327702
6	0.007259	-15.370532	-0.012795
6	-0.080912	-16.742930	0.098208
6	0.649125	-16.943751	-2.207076
1	1.030894	-15.111732	-3.266229
1	-0.407046	-17.219202	1.017713
1	0.913621	-17.581661	-3.043681
6	-0.287767	-14.298911	1.021290
1	-1.323701	-14.350466	1.378214

1	0.354632	-14.398055	1.904873
6	0.662139	-12.080945	-1.801220
1	1.698422	-12.029262	-2.157451
1	0.020603	-11.982481	-2.685623
6	-0.590752	-8.396352	2.478842
1	-1.627964	-8.448930	2.831763
1	0.048514	-8.494084	3.364776
6	0.366463	-6.183277	-0.347674
1	1.403226	-6.132722	-0.702264
1	-0.273039	-6.085924	-1.233449
7	0.168930	-18.946993	-0.909975
6	0.841364	-19.738148	0.019413
6	1.955171	-21.708043	1.609226
6	1.743403	-19.360588	1.012055
6	0.502892	-21.089383	-0.194650
6	1.065995	-22.076977	0.612457
6	2.290077	-20.361490	1.800837
1	2.014092	-18.321006	1.158681
1	0.813147	-23.121840	0.458082
1	2.995407	-20.093196	2.581407
1	2.399987	-22.466792	2.244886
6	-0.600542	-19.780457	-1.719882
6	-1.952077	-21.816101	-3.014926
6	-0.421785	-21.116285	-1.308016
6	-1.459594	-19.448001	-2.765331
6	-2.127275	-20.481885	-3.404063
6	-1.104076	-22.137926	-1.967357
1	-1.604654	-18.416563	-3.066240
1	-2.801881	-20.249231	-4.222302
1	-0.974479	-23.171535	-1.660177
1	-2.488377	-22.601674	-3.537316
6	0.175641	2.911685	1.665963
6	0.335182	5.722954	1.902132
6	-0.139019	3.748952	0.559975
6	0.583550	3.548438	2.872031
6	0.667728	4.917572	2.997135
6	-0.066242	5.111246	0.685846
1	-0.432189	3.290261	-0.373872

1	0.880688	2.941814	3.718096
1	0.998499	5.359933	3.931977
6	0.310780	7.142986	1.703494
6	-0.085478	7.432914	0.431094
6	-0.110982	8.860086	0.227437
6	0.034463	11.613828	0.450015
6	-0.442840	9.635702	-0.889703
6	0.291303	9.467306	1.444958
6	0.366355	10.841014	1.565775
6	-0.366355	11.008078	-0.767630
1	-0.749589	9.167447	-1.820899
1	0.673713	11.308985	2.497028
6	0.007257	13.048092	0.241056
6	-0.382490	13.331783	-1.023073
6	-0.404296	14.769751	-1.228300
6	-0.247652	17.535311	-1.008624
6	-0.720173	15.560159	-2.327702
6	-0.007259	15.370532	-0.012795
6	0.080912	16.742930	0.098208
6	-0.649125	16.943751	-2.207076
1	-1.030894	15.111732	-3.266229
1	0.407046	17.219202	1.017713
1	-0.913621	17.581661	-3.043681
6	0.287767	14.298911	1.021290
1	1.323701	14.350466	1.378214
1	-0.354632	14.398055	1.904873
6	-0.662139	12.080945	-1.801220
1	-1.698422	12.029262	-2.157451
1	-0.020603	11.982481	-2.685623
6	0.590752	8.396352	2.478842
1	1.627964	8.448930	2.831763
1	-0.048514	8.494084	3.364776
6	-0.366463	6.183277	-0.347674
1	-1.403226	6.132722	-0.702264
1	0.273039	6.085924	-1.233449
7	-0.168930	18.946993	-0.909975
6	-0.841364	19.738148	0.019413
6	-1.955171	21.708043	1.609226

6	-1.743403	19.360588	1.012055
6	-0.502892	21.089383	-0.194650
6	-1.065995	22.076977	0.612457
6	-2.290077	20.361490	1.800837
1	-2.014092	18.321006	1.158681
1	-0.813147	23.121840	0.458082
1	-2.995407	20.093196	2.581407
1	-2.399987	22.466792	2.244886
6	0.600542	19.780457	-1.719882
6	1.952077	21.816101	-3.014926
6	0.421785	21.116285	-1.308016
6	1.459594	19.448001	-2.765331
6	2.127275	20.481885	-3.404063
6	1.104076	22.137926	-1.967357
1	1.604654	18.416563	-3.066240
1	2.801881	20.249231	-4.222302
1	0.974479	23.171535	-1.660177
1	2.488377	22.601674	-3.537316

References

[1] X. Zhu, H. Tsuji, J. T. López Navarrete, J. Casado, E. Nakamura, *J. Am. Chem. Soc.*, **2012**, *134*, 19254.
